# Multi-locus DNA metabarcoding of zooplankton communities and scat reveal trophic interactions of a generalist predator

**DOI:** 10.1038/s41598-018-36478-x

**Published:** 2019-01-22

**Authors:** E. L. Carroll, R. Gallego, M. A. Sewell, J. Zeldis, L. Ranjard, H. A. Ross, L. K. Tooman, R. O’Rorke, R. D. Newcomb, R. Constantine

**Affiliations:** 1 School of Biological Sciences, University of Auckland, Auckland, New Zealand; 2 National Institute of Water and Atmospheric Research, Christchurch, New Zealand; 3 Research School of Biology, the Australian National University, Canberra, ACT Australia; 4 The Institute for Plant & Food Research, Auckland, New Zealand; 5 Institute of Marine Science, University of Auckland, Auckland, New Zealand

## Abstract

To understand the ecosystem dynamics that underpin the year-round presence of a large generalist consumer, the Bryde’s whale (*Balaenoptera edeni brydei*), we use a DNA metabarcoding approach and systematic zooplankton surveys to investigate seasonal and regional changes in zooplankton communities and if whale diet reflects such changes. Twenty-four zooplankton community samples were collected from three regions throughout the Hauraki Gulf, New Zealand, over two temperature regimes (warm and cool seasons), as well as 20 samples of opportunistically collected Bryde’s whale scat. Multi-locus DNA barcode libraries were constructed from 18S and COI gene fragments, representing a trade-off between identification and resolution of metazoan taxa. Zooplankton community OTU occurrence and relative read abundance showed regional and seasonal differences based on permutational analyses of variance in both DNA barcodes, with significant changes in biodiversity indices linked to season in COI only. In contrast, we did not find evidence that Bryde’s whale diet shows seasonal or regional trends, but instead indicated clear prey preferences for krill-like crustaceans, copepods, salps and ray-finned fishes independent of prey availability. The year-round presence of Bryde’s whales in the Hauraki Gulf is likely associated with the patterns of distribution and abundance of these key prey items.

## Introduction

Determining trophic interactions is essential for effectively understanding and conserving both species and ecosystems in the face of anthropogenic pressures^1,2^. A key question is whether trophic dynamics are controlled by the availability of resources (‘bottom-up’) or the impact of predation (‘top-down’)^3,4^. For example, zooplankton communities are hypothesised to impact higher trophic levels due to bottom up effects e.g.^5^. That is, the productivity of the food web is controlled by the productivity of the phytoplankton primary producers, which in turn is driven by oceanographic or biogeochemical cycles^6^.

More recently, top down effects are becoming recognised as important moderators of marine ecosystem dynamics^7^. Much work has focused on the impact of krill predators on nutrient cycling in the Southern Ocean, where the availability of iron controls primary productivity^8^. Whale faecal matter is iron rich, and it leaches iron particles into the surface level of the ocean over a 12 hour period^9^. By keeping iron at the surface, the ‘whale pump’ of nutrients found in cetacean scat is hypothesised to enhance productivity and prey abundance, and aids in the transfer of nutrients from areas of high to low productivity^10^. In turn, this is thought to increase both the abundance of krill and the Southern Ocean’s carrying capacity for baleen whales^11,12^. This idea has been invoked to explain the link between primary productivity and abundance of both krill and baleen whales in ice-free areas between the Antarctic Peninsula and South Georgia^11^. In addition, baleen whales can exert strong pressure on marine communities through direct predation, physical engineering and whale falls (for a review see^13^).

**Figure 1:**
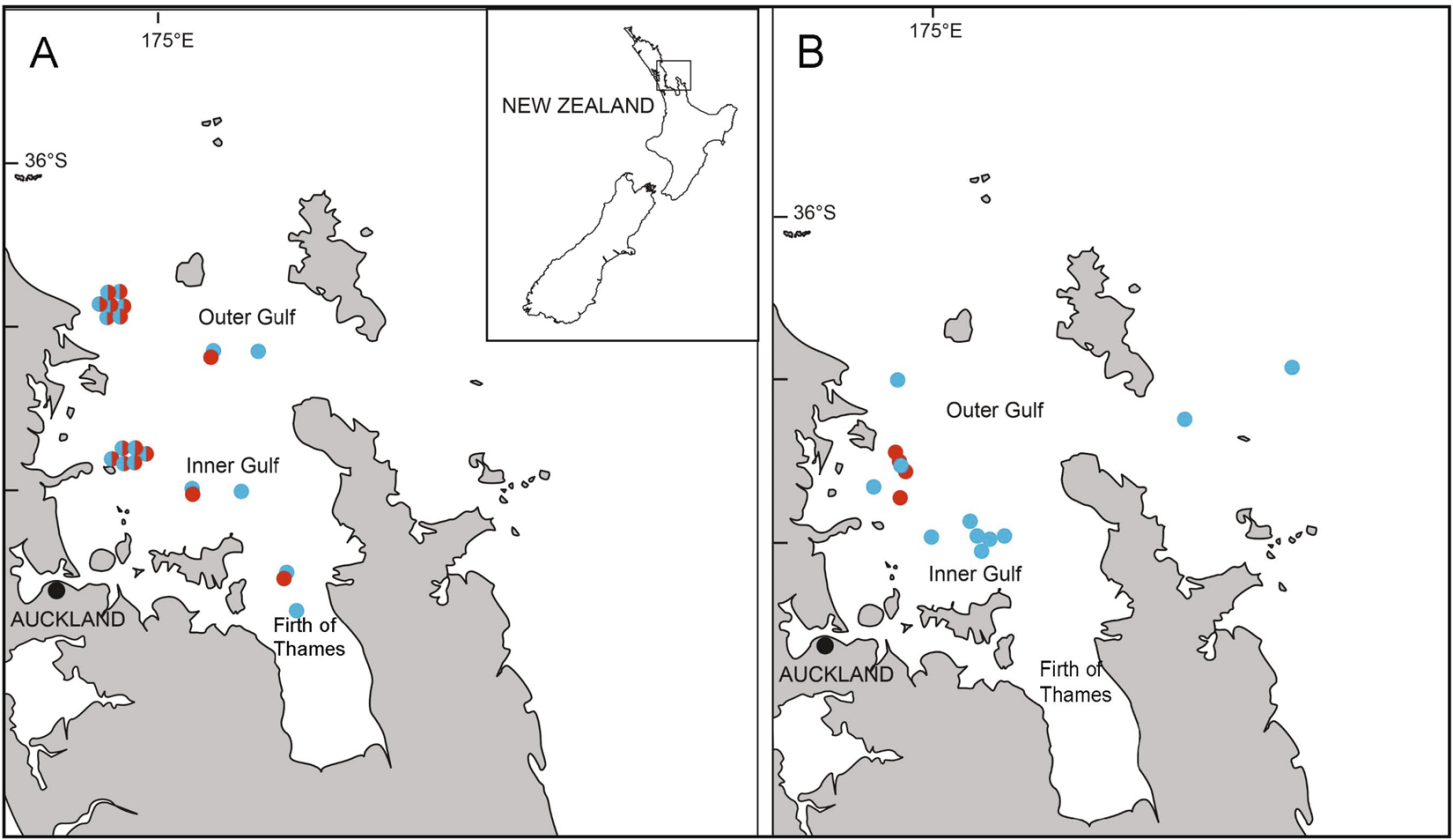
Sampling location and water temperature season of (**A**) zooplankton community samples and (**B**) whale scat samples. The blue circles represent samples collected in the cool water temperature season (winter and spring) and the red circles represent samples collected in the warm temperature season (summer and autumn).

Most studies examining the role of whales as top down regulators have focused on migratory baleen whale populations in polar-regions where iron is the limiting factor e.g.^10^. In some coastal regions, such as the Gulf of Maine, U.S.A., and parts of the Hauraki Gulf, New Zealand (Fig. 1), nitrogen is thought to be a key limiting nutrient for phytoplankton growth^12,14^. In the Gulf of Maine, whales that visit seasonally to feed on plankton and fish are estimated to contribute more nitrogen to the system than all the local rivers combined and may extend the seasonal plankton productivity by effectively recycling nutrients^12^. For the few regions with year-round resident populations of baleen whales, such seasonal effects may be less pronounced and research is lacking on the interaction between resident whales and their prey.

We seek to characterise the trophic dynamics that underpin the resident population of Bryde’s whales *Balaenoptera edeni brydei*^15^ in the Hauraki Gulf. Bryde’s whales are unusual in comparison to other baleen whales in two primary ways. First, several populations of Bryde’s whale, including that found in the Hauraki Gulf, do not migrate to the polar regions to feed during summer months. Instead, the species takes advantage of a range of prey within tropical and temperate waters year round^16^. Second, Bryde’s whales feed across trophic levels including herbivorous and carnivorous zooplankton as well as fish, unlike many other baleen whales that feed predominantly on either large zooplankton or small, schooling fishes^16,17^. However, despite the capacity for generalist feeding behaviours, different sub-populations of Bryde’s whales within South African waters specialise on different prey items: either only euphausiids, only fish or a mixture of both^18^. With the potential for diet flexibility, recent evidence of shifts in preferred prey across seasons in Bryde’s whales in the North Pacific is of interest^19^. Therefore, as a generalist predator with the ability to specialise, the diet of Bryde’s whales needs to be studied on a population level and over seasons to gain a complete picture of prey preferences and trophic interactions in a given region. In addition, many previous studies have been based on whales killed during hunting, and here we sought to take a non-invasive approach to investigate Bryde’s whale diet in a population that is listed as ‘Nationally Critical’^15^.

Given that Bryde’s whales are a generalist predator, foraging in surface waters^20^, we hypothesise that in the Hauraki Gulf the diet of Bryde’s whales follows seasonal shifts in zooplankton community composition. Zooplankton studies using manual sorting techniques and morphological identification have found that seasonal changes in salinity, water temperature and productivity shape the composition of the entire zooplankton community^21,22^. As the Gulf is shallow (<50 m depth), these broad seasonal trends are likely more important drivers of ecosystem dynamics than the limited impact of shorter-term factors such as diurnal variation in environmental conditions^22^.

To understand the ecosystem dynamics that underpin the year-round presence of a large, generalist consumer, the Bryde’s whale, we have used a multi-locus DNA metabarcoding approach and systematic surveys to determine whale diet and prey availability. Metabarcoding provides a powerful method for linking organisms across food webs through DNA-based diet analysis^23^. This is useful in marine environments, as studying the diet and trophic interactions of large, free-ranging species can be challenging. Direct observations of feeding are rare, especially when the predator is evasive or migratory. Stomach content analysis has been successful in some large marine species e.g.^24^; however, manipulation or lethal sampling is not always possible if the species is endangered, large and/or difficult to catch. Stable isotope analysis can provide information on trophic interactions, but is less effective in generalist predators due to the wide variance in isotopic signatures associated with feeding across trophic levels^25^. Diet analysis using DNA techniques offers a useful alternative or compliment to these traditional approaches and has been employed extensively in larger marine species that defecate on land [e.g., penguins and seals^26,27^]. It has also been used to analyse the gut contents or faeces of captive marine specimens^28^, and in studies where the gut contents can be comprehensively sampled, typically in smaller marine organisms^29,30^. The application of DNA techniques on the scat collected from free-ranging mammals offers a direct approach to assess the diet of these generalist predators foraging across multiple trophic levels^31–33^. We also chose to take a multi-locus approach as investigations that use multiple DNA barcodes have an enhanced ability to describe community composition^34^ and species richness^35^ compared with single locus studies, by reducing the biases associated with single DNA barcodes.

Specifically, we address whether DNA metabarcoding can detect the seasonal and geographic changes in zooplankton diversity. We then use this baseline information to test whether the composition of whale diet reflects the observed changes in the zooplankton community or whether the Bryde’s whales of the Hauraki Gulf have a preferred diet.

## Materials and Methods

### Sample collection – zooplankton community samples

To capture a representative sample of zooplankton biodiversity, we sampled the zooplankton community every six weeks from October 2012 to September 2013 at two sites. The first site represented the Inner Gulf (Shearer Rock: 36°38.818S, 174°55.360 E, *n* = 7) and the second site represented the Outer Gulf (Jellicoe Channel: 36°16.024S, 174°55.485 E: Fig. 1A, *n* = 8). Samples were collected using a 30 m vertical haul and a 150 μm mesh standard cod-end plankton net. We augmented this dataset with samples representing a broader geographic region, including the Outer Gulf, Inner Gulf and Firth of Thames collected in October 2012, March 2013 and July 2013 (Research Voyage Programmes KAH1209, KAH1304 and KAH1306 undertaken by the National Institute of Water and Atmospheric Research (NIWA), Fig. 1). Zooplankton were sampled (*n* = 9) using a single vertical haul using 200 μm mesh plankton net for details see^22^. We assigned the samples to align with seasonal temperature regimes: cool-season (Austral winter and spring; 13–17 °C) and warm-season (Austral summer and autumn: 17–21 °C)^36^.

Zooplankton samples were preserved in 100% ethanol and stored at −20 °C until DNA extraction. Zooplankton samples were then divided into sixteenth fractions using a Folsom plankton splitter. The fractions were transferred to a sterile 50 mL tube and topped up to 35 mL with ethanol if required. The tubes were spun at maximum speed (3500 g) for 15 minutes. The resulting zooplankton pellet was transferred to a sterile 2 mL tube that contained two sterile 5 mm metal beads and was homogenised using a Retsch oscillating miller (2 min at 25 Hz). DNA was extracted from the homogenate of each sample using the PowerMax® Soil DNA isolation kit (MOBIO).

### Sample collection – whale scat

To determine whale diet, whale scat samples were opportunistically collected with a 150 μm mesh net between 2011 and 2013 in the Hauraki Gulf (Fig. 1B) and supplemented with an additional four scat samples archived at the University of Auckland^37^. Samples were collected rapidly after a defecation was sighted, with surface waters scooped to collect the visible scat before it sank. All samples were preserved in 100% ethanol and stored at −20 °C. To mitigate contamination risk, samples were analysed in a laboratory where marine research had not been conducted previously. DNA was extracted in a laminar flow UV hood using the QIAamp DNA stool minikit (Qiagen).

### Multi-locus DNA metabarcode library preparation

We constructed multi-locus DNA barcode libraries using 18S and COI gene fragments that represent a trade-off between identification and resolution of metazoan taxa. We targeted the 18S rRNA hypervariable v9 region, with a highly conserved primer binding site that preferentially amplifies across all metazoan phyla^30^. This short fragment has been used in a range of published zooplankton studies, allowing the identification of many operational taxonomic units (OTUs), albeit to a coarse taxonomic resolution (Class or Order)^27,30^. In contrast, COI gives greater taxonomic resolution and biodiversity statistics derived from these genes may be more sensitive measures than those derived from 18S^38^. We chose to use the COI mini-barcode^39^ for whale scat because it has been successful in previous studies and because it amplifies a short fragment length (300–400 bp) from metazoa. Details of PCR primers, amplification and enrichment of prey DNA are described in detail in Supplementary Material 1.1 and Table S1.

We cleaned and quantified amplicons using AMPURE XP magnetic beads (Beckman Coulter) and Qubit dsDNA HS assay kit (Life Technologies), respectively, and then pooled amplicons of different genes from the same sample in equimolar ratios. Nextera index adapters were added to enable identification of individual samples, followed by next-generation sequencing on the Illumina MiSeq platform at New Zealand Genomics.

### Quality control and Operational Taxonomic Unit (OTU) clustering

Sequences were demultiplexed into samples, and samples into DNA barcodes, based on adaptor primer sequences, respectively, using Geneious^40^. We used PEAR^41^ to assemble paired end reads for the COI and 18S DNA barcodes. PEAR also trimmed barcodes and adapters, in addition to implementing a quality control measure of trimming bases with a quality score <25 (equivalent to a base-call having a probability of <1/500 of being incorrect^42^). We used UPARSE to cluster reads into OTUs and to filter singleton OTUs from the dataset^43^. We used 97% clustering threshold based on an initial sensitivity analysis (Supplementary Material 1 and Fig. S1).

### Characterising composition and diversity of zooplankton community and whale scat samples

The NCBI taxonomy database^44^, accessed May 2015, assigned taxonomy to each OTU sequence using custom PERL scripts. The full taxonomy of the top five Blast hits was downloaded and the consensus taxonomy was defined using the program MOTHUR with a consensus confidence threshold of 60% (Blast settings and code found at: https://github.com/LouisRanjard/Plankton_to_pooh). OTUs not identified to Phylum with this threshold were discarded, and subsequent analyses conducted with ‘known’ taxa only. Taxa not identified as marine taxa by the database WORMS were removed; this included likely environmental or laboratory contaminants (e.g. bacteria, mammalian and avian DNA), algae and unicellular eukaryotes^27^ (Table S2). Reads were summarised by Phyla and plotted to visualise the taxonomic composition of the zooplankton community and whale scat samples on a per DNA barcode basis.

To gain more insight into whale diet, we examined all OTUs that were assigned to taxonomic Class with >60% confidence. To show common putative prey species, presence/absence and comparatively high read abundance (>15% reads) of each Class present in scat samples were tabulated. This is to balance concerns that occurrence (presence/absence) summaries can put weight on prey consumed in small quantities and relative read abundance can be impacted by differential recovery of taxa due to technical and analytical limitations (for further information see below and for a recent review see^45^).

We estimated Shannon’s β and γ diversities per DNA barcode, and α diversities per sample, using the R package *vegetarian*^46^, for zooplankton samples and whale scat samples separately. Sample sizes were standardised within each DNA barcode using rarefaction and standard deviations calculated in the same program using 1000 bootstraps.

### Can metabarcoding detect seasonal and regional changes in zooplankton communities?

We hypothesise that whale diet will track the biodiversity and composition of the Hauraki Gulf plankton community. To provide the context for this work, we tested the hypothesis that DNA metabarcoding can detect changes in biodiversity, occurrence and relative abundance of OTUs in the zooplankton community.

#### Biodiversity

To examine changes in biodiversity across time and space, we calculated Shannon’s β and γ diversities per DNA barcode by water temperature-based season (Austral summer/autumn pooled for warm season and Austral winter/spring pooled for cool season) and sampling region (Outer Gulf, Inner Gulf, and Firth of Thames). Data were again rarefied to standard sample sizes and standard deviations calculated using 1000 bootstraps with the R package *vegetarian*.

To test for significant differences in β diversity between season and sampling regions within each sample type, we undertook an analysis of multivariate homogeneity of group dispersions of the sequence data (PERMDISP^47^). As PERMDISP measures the average dissimilarity of individual observations to their group centroid, it has been argued to be a proxy for β diversity as highlighted by^48^. The data were transformed with Gower distances in the R package *vegan*^49^, reduced to principal coordinates and distances between group members and group centroids were calculated. To test if one group was significantly more variable than another, and hence harboured more diversity, we undertook analysis of variance (ANOVA) of the distances to group centroids in R.

#### OTU composition and relative abundance

Analysis of OTU abundance table data is a much debated topic^45,50^. The number of sequence reads per OTU should, at best, be taken as semi-quantitative. The reason for this is that although a component of amplicon abundance is determined by the biomass of organisms in the environment, it is also co-determined by other factors such as the fact that universal DNA markers (such as mitochondrial genes and the nuclear ribosomal genes) can be in greater density in the tissues of some organisms than others^23,51^ and also stochastic factors that occur during DNA extraction, PCR and sequencing^52,53^. Consequently, the OTU-abundance tables in the present study were transformed to reduce the impact of relative read abundance between OTUs on subsequent analyses and using distance measures that provide complimentary information about community composition^45^. To get an estimate of OTU occurrence we reduced the data to a binary (presence/absence) matrix to entirely eliminate the influence of abundance on analyses and then calculated the Jaccard dissimilarity index between samples^54^. To get an estimate of relative read abundance (RRA), we pre-treated the data by 4th root transformation, to downplay the influence of abundance on analyses, and calculated the Bray Curtis dissimilarity index between samples^55^. Both OTU occurrence and RRA measures were calculated using the R package *vegan*.

In addition, we used UniFrac distances^56,57^, to estimate similarity amongst samples by incorporating the phylogenetic distances between OTUs. This gives insight into the phylogenetic variability or taxonomic breadth of the samples, which is distinct from the previously described analyses that are standard ecological tools based on the number of unique OTUs but that do not explicitly consider genetic distinctiveness. To do this, we aligned OTUs in QIIME^58^ using MUSCLE^59^ and constructed a phylogenetic tree from the alignment in QIIME using FastTree^60^, employing the midpoint method. We then calculated weighted and unweighted UniFrac distances, which are more sensitive to changes in abundant and rare taxa, respectively, using the R package *GUniFrac*^61^. Transformations were conducted independently for each DNA barcode (18S, COI) and the resulting data were visualised using principal coordinate analysis (PCO). This unconstrained ordination allows for assessment of any emergent patterns without *a priori* hypotheses.

We tested for significant seasonal (cool and warm) and within-Gulf regional variation using a two-way permutation analysis of variance (PERMANOVA), using the R package *vegan*, with season and region modelled as fixed factors. To test whether the differences detected by PERMANOVA were due to differences in the dispersion from the group centroid or differences between factors, we conducted a PERMDISP analysis based on the underlying similarity matrices used in the PERMANOVA. For those factors (season, region) for which there was not a statistically significant dispersion effect, we undertook a canonical analysis of principal coordinates (CAP)^62^ to establish the distinctiveness of each factor level, using the R package *vegan*. CAP analyses are constrained ordination that can be used when an *a priori* hypotheses concerning differences among groups is suspected, in this case, as highlighted by a significant PERMANOVA result. CAP analysis is useful as it is compatible with any measure of dissimilarity and can detect ecologically important patterns that can be masked by unconstrained ordination procedures^62^.

### Does whale diet reflect changes in the zooplankton community?

#### Seasonal and temporal changes in zooplankton community diversity, abundance or composition

To investigate whether the patterns seen in the zooplankton community were seen in whale diet, we repeated the above analyses using the whale scat sample data, for 18S and COI independently.

#### Is whale diet enriched for particular prey species?

To test the hypothesis that the scat samples were enriched for particular prey species, compared with the general zooplankton samples, we conducted several analyses. First, we constructed similarity matrices corresponding to OTU occurrence, RRA and phylogenetic distances for both the COI and 18S DNA barcodes. We then tested whether sample type explained variation in the dataset, in addition to season and region, using a three-way PERMANOVA.

Second, we tested whether the scat samples were more phylogenetically clustered than the zooplankton community samples, reflecting enrichment of particular prey taxa by calculating Faith’s phylogenetic diversity index for zooplankton and scat samples, for the COI and 18S DNA barcodes. These values were compared with the null values for each sample expected by chance, given the underlying community dataset and phylogenetic trees, generated using the R package *picante*^63^.

## Results

All 24 zooplankton community samples were successfully amplified for the COI and 18S DNA barcodes. Of the 20 whale scat samples, 16 were successfully amplified for both the COI and 18S DNA barcodes.

The MiSeq run produced over 14 million paired end reads. The quality controlled dataset included 2.4 million reads representing 6156 OTUs for 18S, and 3.4 million reads representing 8319 OTUs for COI. We limited our analyses to known zooplankton taxa and discarded reads that were not identified to Phylum with >60% confidence. This dataset included 652,057 reads representing 1,235 OTUs for COI and 1,013,722 reads representing 282 OTUs for 18S (see Table 1 and Table S1 for details by sample type).

**Table 1:** Diversity statistics summarised by season (warm – summer, autumn; cool – winter, spring) and region (Firth of Thames, Inner Gulf and Outer Gulf) for zooplankton samples.

Plankton	COI						18S					
	Total	Warm	Cool	Firth	Inner	Outer	Total	Warm	Cool	Firth	Inner	Outer
n	24 (23)	11 (10)	13 (13)	3 (3)	10 (10)	11 (10)	24 (24)	11 (11)	13 (13)	3 (3)	10 (10)	11 (11)
Reads	153387	74691	78316	31430	55661	65916	112207	60416	51791	14164	39808	58235
OTU_N_	664	373	590	165	414	535	156	125	97	57	82	128
OTU_R_	527	279	452	109	317	412	139	114	85	50	74	113
β-Sh ± sd	4.97 ± 0.03	2.86 ± 0.02	3.76 ± 0.03	2.31 ± 0.02	3.59 ± 0.03	3.59 ± 0.03	3.72 ± 0.02	3.16 ± 0.02	2.71 ± 0.01	1.41 ± 0.01	3.05 ± 0.02	2.93 ± 0.01
γ-Sh ± sd	122.95 ± 1.26	62.71 ± 0.80	102.13 ± 1.11	37.22 ± 0.93	70.28 ± 1.00	127.63 ± 1.70	10.65 ± 0.06	10.58 ± 0.09	6.79 ± 0.06	4.69 ± 0.02	9.32 ± 0.07	7.57 ± 0.08
**Scat**	**COI**						**18S**					
	**Total**	**Warm**	**Cool**	**Firth**	**Inner**	**Outer**	**Total**	**Warm**	**Cool**	**Firth**	**Inner**	**Outer**
n	16 (15)	4 (4)	12 (11)	0	7	7	15 (13)	5 (5)	10 (8)	0	10 (8)	3 (3)
Reads	230677	117117	112885	—	60556	155840	649334	128832	519762	—	462701	43517
OTU_N_	810	426	702	—	524	557	228	138	216	—	212	134
OTU_R_	558	248	499	—	377	348	142	86	129	—	126	75
β-Sh ± sd	6.13 ± 0.05	3.99 ± 0.01	5.12 ± 0.04	—	4.13 ± 0.04	3.69 ± 0.03	3.70 ± 0.02	2.95 ± 0.02	2.91 ± 0.02	—	3.12 ± 0.02	1.89 ± 0.01
γ-Sh ± sd	185.25 ± 1.63	93.73 ± 1.54	169.52 ± 1.91	—	123.91 ± 1.63	15.47 ± 1.59	16.72 ± 0.14	12.11 ± 0.16	14.00 ± 0.16	—	15.74 ± 0.17	8.10 ± 0.15

Statistics are shown by DNA barcode, sample type and include: n; sample size, (n); sample size used to calculate rarefaction-based statistics, Reads; total number of reads, OTU_N_; total number of OTUs, OTU_R_; rarefied number of OTUs, β; Shannon’s β (β – Sh) and γ (γ – Sh) diversity index, with estimated standard deviations (±sd). Rarefied number of OTUs, β and γ diversity statistics were estimated using 952 reads for COI and 1956 reads for 18S.

### Characterising the composition of zooplankton samples

**Figure 2:**
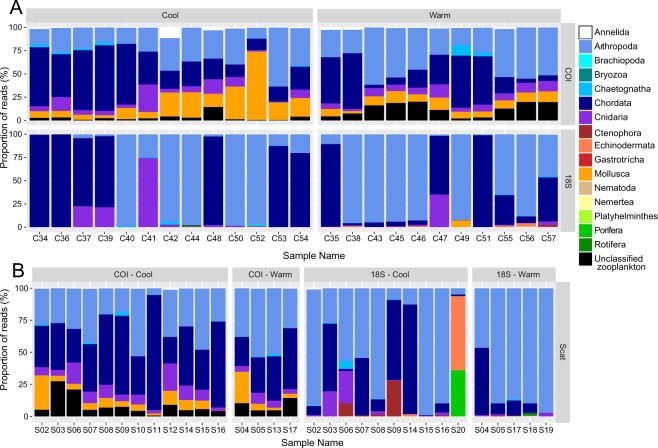
Summary of reads by taxa stratified by season (cool – winter, spring; warm – summer, autumn) and DNA barcode (COI, 18S) for A. zooplankton community and B. scat samples.

All 24 zooplankton community samples produced reads for each DNA barcode (Table S3). Arthropoda and Chordata were the most common Phyla across all the samples for the COI and 18S DNA barcodes (Fig. 2). The COI DNA barcode showed the highest γ-level diversity (Table 1), with Arthropoda, Mollusca, Echinodermata and Chordata all common.

### Characterising the composition of whale scat

All scat samples produced reads for either COI and/or 18S DNA barcodes, with the exception of sample S01 (Table S4). Two scat samples (S16 and S20) in the 18S DNA barcode were excluded from rarefaction-based analyses due to the low number of reads. Two other scat samples were excluded from the regional biodiversity and PERMANOVA analyses (S17 and S02) due to a lack of location data (see Table S4). One scat sample was excluded from the COI DNA barcode dataset due to a low number of reads (S02, Table S4, <1000 reads). Therefore, the total number of samples that were used in analyses and that had associated GPS location data was 13 for COI and 14 for 18S, respectively.

Chordata and Arthropoda were the most common Phyla observed across both DNA barcodes (Fig. 2). We restricted our analyses to the 1,101 COI DNA barcode OTUs and the 267 18S DNA barcode OTUs identified to Class. In an OTU occurrence analysis, the most common taxa in the scat (both COI and 18S DNA barcodes) were krill (Arthropoda:Malacostraca), copepods (Arthropoda:Maxillopoda), arrow worms (Chaetognatha:Sagittoidea), fishes (e.g. Chordata:Actinopterygii), salps (Chordata:Thaliacea) and jellyfish-like hydrozoans (Cnidaria: see Table 2). The 18S barcode also highlighted free-swimming tunicates (Chordata:Appendicularia) and comb jellies (Ctenophora:Tentaculata) as potentially important prey species, while planktonic arthropods (Arthropoda:Chelicerata) and crustaceans (Arthropoda:Branchiopoda) were identified in the COI dataset. In the RRA analysis (>15% of reads), copepods, krill, salps, ray-finned fishes, and planktonic crustaceans were well represented.

**Table 2:** The frequency of putative prey taxa occurring across the whale scat samples, identified to Phylum and Class.

Phylum	Class	Common name	Presence/Absence		High Abundance	
			18S	COI	18S	COI
Annelida	Clitellata	marine worm	3	12	0	0
	Polychaeta	marine warm	7	0	0	0
Arthropoda	Branchiopoda	planktonic crustaceans	1	16	0	5
	Chelicerata	planktonic arthropods	3	13	0	0
	Chilopoda		0	8	0	0
	Malacostraca	krill	15	16	6	1
	Maxillopoda	copepods	15	16	7	3
Chaetognatha	Sagittoidea	arrow worms	11	12	0	0
Chordata	Actinopterygii	ray-finned fish	13	16	2	3
	Appendicularia	tunicates	12	0	0	0
	Elasmobranchii	cartilaginous fish	0	2	0	0
	Holocephali	cartilaginous fish	0	5	0	0
	Sarcopterygii	lobe-finned fishes	0	8	0	0
	Thaliacea	salps	14	16	4	15
Cnidaria	Hydrozoa	jellyfish-like	13	16	2	2
	Scyphozoa	jellyfish-like	0	1	0	0
Ctenophora	Tentaculata	comb jellies	12	0	1	0
Echinodermata	Asteroidea	echinoderms	0	4	0	0
	Echinoidea	echinoderms	4	0	1	0
Gastroricha	Chetonotida	echinoderms	1	0	0	0
Mollusca	Bivalvia	bivalves	2	10	0	0
	Gastropoda	seas snails	3	16	0	2
Nematoda	Chromadorea	marine roundworm	1	0	0	0
Nemertea	Enopla	marine worm	2	0	0	0
Platyhelminthes	Trematoda	flatworm	1	0	0	0
	Turbellaria	flatworm	4	0	1	0
Porifera	Demospongiae	sponge	3	0	0	0
Rotifera	Eurotatoria	zooplankton	2	0	0	0
Zooplankton	unclassified	zooplankton	0	16	0	3

Data are shown as both presence/absence and relatively high read abundance taxa (>15% reads per sample). Total sample sizes were 15 for 18S and 16 for COI.

### Testing the hypothesis that metabarcoding can detect seasonal and regional changes in zooplankton

#### Metabarcoding detects differences in biodiversity of zooplankton communities between seasons and regions

Diversity statistics for season and region for the zooplankton community samples are summarised in Table 1 and Table S3. PERMDISP analysis of the zooplankton community samples indicated a significant effect of season on the level of β diversity in the COI DNA barcode (Tables 1 and 3). Cool season samples (e.g. Shannon diversity statistics: COI β = 3.76, γ = 102.13) typically had higher levels of diversity compared with the warm season samples (COI β = 2.86, γ = 62.71: Table 1). In addition, there was a significant effect of region on the level of β diversity using the COI DNA barcode (Table 3), with the highest diversity in the Outer Gulf and decreasing levels with increasing proximity to the Firth of Thames (e.g. Shannon diversity statistic for COI: Outer Gulf β = 3.59, γ = 127.63; Inner Gulf β = 3.59, γ = 70.28; Firth of Thames, β = 2.31, γ = 37.22). The 18S DNA barcode did not show any significant differences in diversity with either season or region (Tables 1 and ANOVA section of Table 3).

**Table 3:** Significance of tests to detect differences in zooplankton community and whale scat samples using different underlying distance matrices.

Zooplankton	Season							Region						
	ANOVA			PERMANOVA				ANOVA			PERMANOVA			
	D.F.	*F*-value	*p-*value	Bray	Jaccard	Uni-U	Uni-W	D.F.	*F*-value	*p*-value	Bray	Jaccard	Uni-U	Uni-W
COI	1, 21	5.249	0.042	<0.001	0.001	<0.001	0.025	2, 20	3.716	0.039	0.004	0.003	0.003	0.270
18S	1, 22	0.353	0.559	0.032	0.047	0.032	0.005	2, 21	0.813	0.457	0.005	0.006	0.014	0.008
**Whale scat**														
COI	1, 13	0.415	0.484	0.716	0.716	0.443	0.519	1, 12	0.003	0.776	0.114	0.086	0.041	0.550
18S	1, 11	0.607	0.452	0.331	0.336	0.516	0.152	1, 9	0.037	0.851	0.229	0.322	0.476	0.651

ANOVA: D.F. reflects the degrees of freedom (between and within group), *F*-value and *p*-value of the ANOVA of the multivariate homogeneity of groups’ dispersions to test for significant differences in β diversity between season (cool or warm) and regions (Firth, Inner and Outer Gulf for zooplankton; Inner and Outer Gulf for whale scat and matched water control). PERMANOVA reflects the significance of multi-factorial (Season and Region) permutational multivariate analysis of variance, using different underlying data distance matrices reflecting OTU occurrence (Jaccard distance on presence/absence transformed data (Jaccard) and unweighted (Uni-U) UniFrac distance matrices) and relative read abundance (Bray-Curtis distance on fourth root transformed data (Bray); and weighted (Uni-W) UniFrac distance matrices).

#### Metabarcoding detects differences in OTU occurrence and relative read abundance of zooplankton communities between seasons and regions

**Figure 3:**
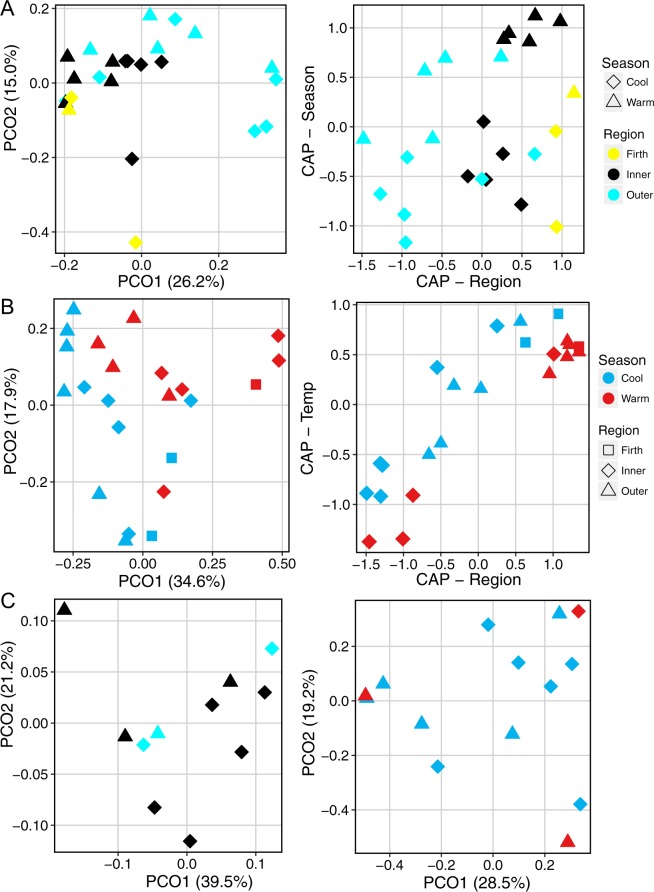
Clustering of zooplankton community and whale scat taxa by OTU occurrence and relative read abundance visualised using principal coordinate analysis (PCO) and canonical analysis of principal coordinates (CAP). (**A**) Visualisation of PCO (left) and CAP (right) analyses of zooplankton community sample using the unweighted UniFrac distance matrix on 18S DNA barcode data, with samples colour coded by sampling region and; (**B**) Visualisation of PCO (left) and CAP (right) analyses of zooplankton community samples using Bray-Curtis distance matrix on fourth root transformed COI DNA barcode data, with samples colour coded by sampling season (**C**). Visualisation of PCO analyses of whale scat samples clustered by unweighted UniFrac distance matrix of 18S DNA barcode data, with samples colour coded by sampling region (left), and Bray-Curtis distance matrix of fourth root transformed COI DNA barcode data, with samples colour coded by sampling season (right).

Principal coordinate analyses showed a trend towards regional and seasonal differences in the zooplankton samples in both DNA barcodes, regardless of the transformation employed (Fig. 3; Supplementary Material 1.2). Multi-factorial PERMANOVA analyses found that the differences in zooplankton community linked to season and region were statistically significant for OTU occurrence, RRA and phylogenetic measures for both DNA barcodes (Table 3). There were no significant interactions between factors in the PERMANOVA analyses and there was no evidence of differences dispersion between factors, based on the PERMDISP test.

A CAP analysis allows for better discrimination of regions and/or seasons by finding axes in principal coordinate space that best discriminate amongst *a priori* groups. CAP analysis of both DNA barcodes showed that OTU occurrence, RRA and phylogenetic measures discriminated zooplankton samples based on sampling region on a gradient from the Outer Gulf to the Firth of Thames. This is shown visually in Fig. 3A, which shows the samples cluster broadly by region using the PCO of the 18S data transformed using the unweighted Unifrac distance (left panel). The ability to discriminate between sample types is highlighted by the CAP analysis (Fig. 3A, right panel), which shows regions being differentiated using the Regional CAP score (Outer Gulf samples have negative scores, moving to more positive values for Inner Gulf and Firth of Thames) and the Season CAP score (generally cool water samples have negative values and warm water season samples have positive values). However, there was some overlap between the sampling regions in the CAP analyses, particularly between the Inner Gulf and the Firth of Thames (e.g. Fig. 3).

CAP analyses also showed samples clustered by season with some overlap for the COI DNA barcode (Fig. 3) but less so for the 18S DNA barcode (Supplementary Material 1.2). This is shown in Fig. 3B, where, using the COI DNA barcode transformed to reflect RRA of OTUs (Bray Curtis distance matrix), samples cluster broadly by season temperature with the PCO analyses (red: warm temperature season; blue: cool temperature season), but show more discrimination with the CAP analysis. Specifically, cool season samples cluster on upper left quadrant and the warm season samples on the lower right quadrant, with mostly negative and positive CAP (Region) scores for the Outer Gulf and Inner Gulf/Firth of Thames samples, respectively (Fig. 3B).

### Testing the hypothesis that whale diet reflects changes in the zooplankton community

#### Whale scat samples did not consistently reflect seasonal and temporal changes in zooplankton diversity, abundance or composition

ANOVA analyses did not indicate any significant effect of season or region on the level of β diversity for the scat, for either the 18S or COI DNA barcodes (Table 3). Furthermore, principal coordinate analyses of similarity matrices indicating sample composition, relative read abundance and phylogenetic similarity did not reveal clustering by season for the whale scat (Fig. 3 and Supplementary Material 1.2). This is shown by comparing the way that the zooplankton samples cluster by season in Fig. 3B with the lack of obvious clustering of the whale scat data in Fig. 3C (right panel), despite both visualisations using the COI DNA barcode data transformed to reflect RRA. Principal coordinate analysis showed some indication that scat samples clustered by region, based on measures that are sensitive to the relative read abundance of taxa for the COI DNA barcode (Unweighted UniFrac and Bray Curtis distance measures, Supplementary Material). The PERMANOVA analyses showed no significant effect of either region or season on the scat, based on the OTU occurrence, RRA and weighted phylogenetic distance measures. In contrast, the unweighted UniFrac distance measure that reflects rare taxa showed a significant effect by region on the scat samples (COI barcode; p = 0.041, Table 3): CAP analyses of this distance matrix with region as a factor clearly discriminated the Inner and Outer Gulf scat samples (Supplementary Material 1.2).

#### The communities present in whale scat are substantially different from those in zooplankton community samples

**Figure 4:**
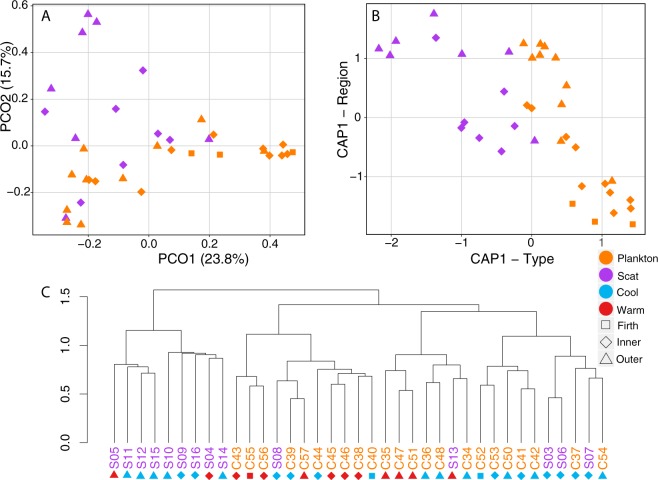
Clustering of zooplankton community and whale scat samples using (**A**) principal coordinate and (**B**) canonical analysis of principal coordinates (CAP) analyses of the COI DNA barcode, based on a Jaccard distance matrix on presence/absence transformed data. (**C**) Dendrogram of samples clustered using the unweighted UniFrac distance of the COI DNA barcode.

Principal coordinate analysis suggested that the scat and zooplankton samples clustered into groups representing sample type, with some overlap (Fig. 4, Supplementary Material 1.2). When overlap occurred, the scat samples often clustered with zooplankton community samples from the same region (e.g. Fig. 4C). PERMANOVA analyses showed significant differences in zooplankton OTU occurrence, RRA and phylogenetic distances between sample types, season and regions, using both the 18S and COI barcodes (Table S5). CAP analysis showed clear clustering by sample type, using both the COI and 18S DNA barcodes, regardless of the underlying distance matrix (Fig. 4, Supplementary Material 1.2).

A greater number of scat samples (70%: 9/13) than zooplankton samples (33%: 8/24) were more clustered than expected using 18S DNA barcode, based on Faith’s phylogenetic diversity statistic (Supplementary Material 2). However, most scat (93%: 13/14) and all (100%: 23/23) zooplankton samples were more clustered than expected under the null model, based on the COI barcode (Supplementary Material 2).

## Discussion

We have employed multi-locus DNA metabarcoding to examine seasonal and regional trends in the zooplankton community of the Hauraki Gulf, New Zealand and provide insight into how this trophic level is utilised by a year-round, resident predator, the Bryde’s whale. In contrast to our initial expectation that, as large, generalist consumers with high energetic requirements and the capacity to feed on very different prey (zooplankton and fishes), the species would be opportunistic feeders, we did not find evidence that Bryde’s whale diet follows seasonal or regional trends in zooplankton community diversity. Instead, the species appears to have clear prey preferences independent of potential prey availability, focusing on krill-like crustaceans and copepods year-round, with ray-finned fishes and salps also common prey items (Table 2).

Phylogenetic clustering and multidimensional scaling of the whale scat samples supports the finding of the narrower and distinct focus of Bryde’s whale diet compared with the broader zooplankton community diet. This is consistent with previous research showing seasonal and population level preferences for particular prey items in Bryde’s whales from the North Atlantic^19^ and South Africa^18^.

Bryde’s whales, like other rorqual whales, forage using lunge feeding, whereby the whale engulfs a large volume of prey-filled water and then filters the water through baleen, trapping prey in the mouth^64,65^. Lunge feeding strategies are modulated in response to prey density, agility and depth, to maximise foraging efficiency^66–68^. Little work has been done on the foraging behavior of non-migratory Bryde’s whales [although see^20,69,70^], but we hypothesise that as rorqual whales, the species will show similar efforts to maximise foraging efficiency. Bryde’s whales forage during the day and rest at night, with high activity levels when feeding on surface zooplankton^20^. They will employ different strategies when feeding on copepods or krill-like crustaceans compared with more agile schooling fishes, both of which were shown to be an important part of their diet in this study and in previous work^18,19,37^. The balance between maximising prey capture whilst minimising energy expenditure is an area worthy of further study for Bryde’s whales that may have different energetic constraints than migrating whales^67^.

DNA metabarcoding also identified salps as a potentially important food source for Bryde’s whales in the Hauraki Gulf. This is unexpected as salps have traditionally been considered a nutritionally poor taxa^71^. However, recent evidence shows that salps in the Southern Ocean form an important part of whale and seabird diet with higher levels of protein, carbon content and other nutrients than previously thought^71,72^. *Thalia democratica* is seasonally common in the Hauraki Gulf where it can be found in densities in excess of 300 per m^3^ ^22,73^, and could form an important and regular part of the diet for Bryde’s whales, as it does for other predators^74^. It is unknown whether or how Bryde’s whales are choosing their prey in other populations^16^, but there is evidence that other baleen whales make decisions about prey aggregations before expending energy on a lunge feeding bout^64^. Much previously published diet work on Bryde’s whales has been conducted using lethal sampling and stomach contents analysis, where such small, soft-bodied prey items would likely be unidentifiable e.g.^18,19^. We hypothesise that the whales may switch to lower-energy feeding strategies while targeting these potentially less valuable prey items. Furthermore, it may be that the energetic demands of a non-migratory, resident population living year-round in temperate waters are lower than migratory baleen whales.

The targeting of preferred prey, despite variation in the underlying zooplankton community, highlights how the New Zealand Bryde’s whale population likely relies on certain foods to maintain a year-round presence in the Gulf. If these prey decline, individual Bryde’s whales may leave the Gulf to find food^16^. Such bottom-up effects, where the distribution of prey dictates the distribution of predators, has been seen in the minke whale (*Balaenoptera acutorostrata*) around Scotland in response to sandeel (*Ammodytes* spp.) and herring (*Clupea harengus*) distribution^75^. This is in contrast to prey switching that has been seen over several years to a decade in response to changes in available prey in many baleen whale and toothed whale species in the Barents^76^ and Norwegian Seas^77^. It has even been suggested that the gray whale (*Eschrichtius robustus*), a specialist benthic invertebrate predator, switched foraging strategies to become a more generalist filter feeder during the Pleistocene glacial maxima, enabling the species to maintain its population size across glacial cycles^78^.

As is typical in marine animals, we rarely observe what prey is being consumed by the whales directly, which limits our ability to understand their ecosystem interactions. Therefore, DNA metabarcoding is extremely effective in determining the importance of prey availability, although with the caveat that the present study has a small sample size, reflecting the difficulty in working with large, mobile and endangered marine species. However, we do recommend caution when interpreting zooplankton community data by region as a reliable indicator of whale prey preference because whales often move between the three Gulf regions and based on other baleen whale data, we estimate digestion time spans ~18 hours^79^, rendering any assumption that a scat in one region represents a meal in that region dubious. This is typically not an important consideration for diet analysis for large whales where the majority are not generalist foragers, but instead focus on one prey type. Additionally, the zooplankton samples were collected every 6 weeks from October 2012 to September 2013, when most of the whale scat samples were collected. Therefore it should provide a good reflection of the potential prey environment^22^, but there could be unobserved fluctuations that were missed across time and space. This effect could have been amplified by augmenting the dataset with historically collected scat samples.

DNA-based diet studies using metabarcoding are becoming an increasingly common way to study trophic interactions in both terrestrial [e.g.^80–82^] and marine ecosystems [e.g.^26,29,83^]. The present study utilised DNA derived from assemblages of animals sampled by plankton net and compared these to DNA derived from Bryde’s whale scat. It is highly probable that the context from which DNA is extracted will affect what DNA is amplified and sequenced. For example, scat is dense in gut microbes and the sloughed off cells of the predator that excreted it, but water samples would not be so dense in these. Scat could also contain PCR inhibitors^84^. To minimise how predator DNA influenced the PCR of prey amplicons, the present study used DNA-blocking primers or PNA-clamps to minimise the influence of predator DNA on the polymerisation of prey templates, which is an established approach^83,85^. However, it is conceivable that the addition of these oligonucleotides might have had an influence on the PCR process. In hindsight, we could investigate the impact of adding the blockers to the environmental samples. However, PNA is highly specific, discriminating when there is even a single nucleotide difference between target and non-target^86^ and therefore not likely to interfere with the reaction. DNA-clamps are similarly discriminatory on highly variable loci^26,87^, such as the mitochondrial targets that we use in the present study. If interference from DNA-blockers artefactually caused any difference between scat and plankton samples, then we would anticipate that differences would be greater for organisms that are taxonomically closer to the predator that the DNA-blocker was designed against. This was not the case as various chordates occurred in frequencies and abundances in scat samples that often exceeded that observed in water samples – despite the use of blocking primers targeted at the mammalian predator. Therefore, although it is possible that the different treatment of scat and environmental samples altered the species data matrices analysed in this study, it appears that the results obtained do reflect genuine differences between samples.

Additionally, as discussed in the Methods, relative read abundance of DNA barcodes is at best semi-quantitative, due to factors such as preferential digestion and transit times^88^, followed by biases that are introduced through laboratory protocols and inherent qualities of species, e.g. bias in primer binding, PCR amplification, gene copy number and number of cells between species^23^. Although it is tempting to assume that the abundance of sequencing reads reflects the relative biomass of organisms, some metabarcoding studies have found that read abundance do relate to biomass density whilst others have found this is not necessarily the case^45,50^. Presence/absence data are consequently less subject to potential biases caused by laboratory artefacts and the stochastic density of DNA target loci inside the tissues of organisms. However, presence/absence data sets are at risk of exaggerating the ecological role of organisms with low abundance^45^. This is problematic with a diet study of large megafauna, because organisms that occur in lower abundance are less likely to sustain a resident population of Bryde’s whales. Transformed abundance data were also analysed in the present study (Table 3) and were found to be concordant with the results from presence absence data (Table 3). Therefore, we can state with some confidence although our data are likely to be a distorted in how they represent relative biomass of organisms, they do index the relative importance of particular organisms in the feeding ecology of Bryde’s whales. Although we caution against treating the relative abundances of reads as being precise representations of biomass, there are several recent metabarcoding studies that do report a good correspondence between biomass and proportions of sequencing reads^50^. Sampling whale scat from the marine environment also comes with the risk of concomitant sampling of marine species that are not prey although this was not found to be an issue in our study (Supplementary Material 3).

Furthermore, scat typically contains degraded DNA templates, necessitating the use of small gene fragments. The taxonomic resolution of this approach is a limitation in this and other diet studies. Indeed, quantifying the levels of zooplankton and whale diet community diversity was hindered by a lack of reference DNA barcoding data on marine zooplankton species. This is a concern for the advancement of community ecology research in the marine environment^89,90^, especially when compared to recent terrestrial studies^91^. In contrast to the terrestrial invertebrate databases that are well represented across all taxa, curated databases used in other marine diet studies have a paucity of zooplankton species. Many OTUs for both DNA barcodes that we used could not be assigned to Phylum, despite confidence in the underlying sequence quality. The assigned OTUs are likely to have biases associated with coverage of different Phyla and available sequence information. This problem is partially accounted for by the multi-locus DNA metabarcoding approach, which permits reference datasets across different loci to be utilised for taxa identification. For example, cartilaginous fishes were detected by COI DNA barcode in whale scat, but not the 18S DNA barcode. Given the high diversity within the marine environment, there is a need for researchers to improve the quality of the marine genetic reference databases^89^.

Despite such limitations, advances in methodological and analytical techniques mean that DNA diet studies are becoming increasingly quantitative e.g.^92,93^ and the importance of these methods are demonstrated by the fact they are already being used to aid restoration and relocation plans for endangered species e.g.^94^. For example, genome skimming through PCR-free shot-gun sequencing has been proposed as a step towards a more accurate characterisation of community composition and diversity^95,96^. This could reduce the issues with PCR-based DNA metabarcoding that leads to biased diversity and composition studies^97^. Problems with PCR could bias our results, for example, the finding of region as a significant factor influencing scat sample composition based on unweighted UniFrac distance. This is a measure that reflects changes in rare taxa, and therefore could be influenced by OTUs that appear rare due to poor amplification rate. This is unlikely to have influenced our findings with regards to whale diet, but may impact our interpretation of regional and seasonal diversity, in particular with the use of COI, a standard barcoding gene targeted in only a small proportion of marine metazoans^89^.

### Validation of metabarcoding to quantify zooplankton community diversity and composition

Multi-locus DNA metabarcoding reflected previously described changes in the zooplankton community across temporal and spatial scales in the Gulf^22^. The Outer Gulf, where coastal and shelf waters converge, had the highest level of diversity in the COI DNA barcode. This likely reflects the diversity in the Outer Gulf zooplankton community, as it contains a concomitant mixture of both neritic and oceanic zooplankton assemblages, and communities characteristic of the transitional region^21,22^. In contrast, the Inner Gulf supports a range of seasonally variable neritic fauna, but is less likely to contain oceanic or transitional zooplankton^22^. The similarity in patterns of distribution and diversity between our study and previous work is useful for long-term comparisons of large predator diet and movement patterns.

Both zooplankton community DNA barcodes showed significant differences in biodiversity by season, emphasising the high level of diversity captured in the cool season (winter and spring). Spring is typically when wind-driven, seasonal upwelling occurs in the Gulf, bringing high nutrient waters and facilitating the influx of oceanic species into the Outer Gulf. During the late austral summer (February), these winds subside, allowing the intrusion of the East Auckland Current surface water, and its accompanying high salinity, low nutrient and warmer waters^73,98^. These warm water masses predominate across the Gulf and homogenise zooplankton communities across the regions, leading to lower levels of diversity in the Gulf as a whole, based on previous work^22^ and as highlighted in our study. Understanding the levels of biodiversity across different spatial and temporal scales, and diversity within and between local assemblages is important when determining community ecology processes^99^.

## Conclusion

Large consumers, such as whales, seabirds, sharks and other large predatory fish often exist through niche separation, prey specialisation or large seasonal migrations to areas of high prey abundance. The Bryde’s whale is an atypical baleen whale as it does not migrate to feed on seasonally abundant prey. This and other work show that DNA diet studies are able to identify primary dietary components e.g.^27,100^, providing the ability to non-invasively assess the potential prey and the diet of large, solitary predators that spend most of their life below the sea surface feeding on organisms that are not visible to the naked eye. Despite our initial hypothesis that they were opportunistic, generalist predators, our study suggests that Bryde’s whales in the Hauraki Gulf have preferred prey and may therefore be subject to bottom up trophic pressures. Further comparative ecological studies in the Hauraki Gulf are likely to further resolve prey partitioning using DNA diet methods e.g.^82^ and provide information for mitigating anthropogenic impacts to populations and the broader marine community^101^.

## Electronic supplementary material

Supplementary Information
