# Supplementary material for: Multi-locus DNA metabarcoding of zooplankton communities and scat reveal trophic interactions of a generalist predator
Source: Sci Rep. 2019 Jan 22;9:281. doi: 10.1038/s41598-018-36478-x (PMC6342929; doi:10.1038/s41598-018-36478-x)
Supplement: Supplementary file 1 — Supplementary Information [file 41598_2018_36478_MOESM1_ESM.pdf]

Supplementary Material For:

Multi-locus DNA metabarcoding of zooplankton communities and scat reveal trophic interactions of a generalist predator

Running page head: Metabarcoding of whale trophic interactions

E. L. Carroll[1], R. Gallego[1], M. A. Sewell[1], J. Zeldis[2], L. Ranjard[3], H. A. Ross[1], L. K. Tooman[4], R. O'Rorke[1], R. D. Newcomb[1,4], and R. Constantine[1,5*]

[1.] School of Biological Sciences, University of Auckland, Auckland, New Zealand

[2.] National Institute of Water and Atmospheric Research, Christchurch, New Zealand

[3.] Research School of Biology, the Australian National University, ACT, Australia

[4.] The Institute for Plant & Food Research, Auckland, New Zealand

[5.] Institute of Marine Science, University of Auckland, Auckland, New Zealand

* Corresponding author: e.carroll@auckland.ac.nz

Carroll et al. Supplementary Material 1

Supplementary Material 1.1: DNA barcode library preparation

We constructed DNA barcode libraries comprising 18S and COI gene fragments for the zooplankton community and whale scat samples. We amplified the 18S gene using primers EukB and 18s_v9_Con [1] , modified to have the Illumina Nextera sequencing overhang adapters (Supplementary Table 1), and PCR conditions described previously [1]. To enrich for prey DNA in the whale scat and for consistency in the matched water control, we used a PNA-clamp (CGACCGTCTTCTCAGC-Lys) that binds downstream of PCR primers and arrests polymerisation [1].

We amplified a 300-400 bp fragment of the COI gene using primers jgHCO1298 and mlCOIintF [2] modified to have the Illumina Nextera sequencing primer overhang adapters (Supplementary Table 1). The PCR was conducted in a 25 µL reaction containing 1xKAPA 2G buffer (KAPA Biosystems), 0.25 µM primers, 0.2mM dNTPs, 1mM Mg, and 1 unit of KAPA 2G taq polymerase (KAPA Biosystems). The PCR reaction had an initial denaturation period of two minutes at 95°C, followed by 35 cycles of 95°C for 20s, 48°C for 30s and 72°C for 60s, and a final extension period of three minutes at 72°C. To inhibit the amplification of predator DNA in the whale scat and for consistency in the matched water controls, we added a blocking primer (5'-ATCCCCCTTTAGCCGGAAATCTAG-3's). This was designed by aligning potential prey [sourced from 2] and whale COI gene sequences sourced from genbank together in geneious (Biomatters, available from http://www.geneious.com). The final concentration of blocking primer of 1.25 µM was found through PCR trials of known quantities of predator and prey DNA (data not shown).

Given the degraded DNA template present in the whale scat samples, we undertook several precautions. As previously mentioned, DNA was extracted in a laminar flow UV hood to minimise contamination. All PCR mixtures were prepared in a UV laminar flow hood in a separate room. DNA template was added to the PCR mixture in a separate UV flow hood. Multiple negative controls were used per PCR and results were only used from PCRs were there were no visible bands in the negative controls. Furthermore, sample types were extracted and amplified, and amplicons cleaned, in separate experiments, to limit the chance of cross-contamination.

Selecting clustering threshold

To select the clustering threshold for use in the analyses, we assessed the change in measures of biodiversity with different clustering levels, by calculating the α, β and γ diversity indices (effective and Shannon) of Jost [3,4] using the R package *vegetarian* (Charney and Record 2012) for each DNA barcode. Several series of OTUs were created by clustering at iterative levels of sequence similarity, incrementing by 1% between 90% and 100% [6]. This revealed that clustering at 98-100% similarity resulted in high levels of diversity across all three DNA barcodes (Supplementary Fig. 1). The diversity indices started to plateau when clustering was at 97%. This threshold was used for further analyses.

Table S1: Amplicon library primers.

Gene	Primer	Sequence	Reference
18S	NexF-V9F	TCGTCGGCAGCGTCAGATGTGTATAAGAGACAG TGATCCTTCTGCAGGTTCACCTAC	O'Rorke et al. [1]
	NexR-V9R	GTCTCGTGGGCTCGGAGATGTGTATAAGAGACAG CCCTTTGTACACACCGCCC	
COI	NexF-mlCOIintF	TCGTCGGCAGCGTCAGATGTGTATAAGAGACAG GGWACWGGWTGAACWGTWTAYCCYCC	Leray et al.[2]
	NexR-jgHCO2198	GTCTCGTGGGCTCGGAGATGTGTATAAGAGACAG TAIACYTCIGGRTGICCRAARAAYCA	

Table S2: List of Phyla and their common name that were removed from the analyses due to several factors, including likely being environmental or laboratory contamination (e.g. birds, likely environmental contamination), or because they are not zooplankton taxa (e.g algae).

Category	Phylum	Common Name
Contaminants	Mammalia	mammals
	Aves	birds
	Fungi	fungi
Not zooplankton taxa	Heterokonta	algae
	Haptophyte	algae
	Rhodophyta	algae
	Viridiplantae	algae
	Telonemida	algae
	Cryptophyta	algae
	Bacteria	bacteria
	Arthropoda:Insecta	insects
	Arthropoda:Chiliopoda	millipedes
	Plasmodium	Single-celled eukaryotes
	Myzozoa	Single-celled eukaryotes
	Centroheliozoa	Single-celled eukaryotes
	Choanoflagellida	Single-celled eukaryotes
	Euglenozoa	Single-celled eukaryotes
	Ciliophora	Single-celled eukaryotes
	Rhizaria	Primarily single-celled eukaryotes

Table S3: The number of reads, Shannon (Sh-α) α diversity calculated per zooplankton sample. Diversity statistics were calculated by subsampling to the lowest number of reads per sample per DNA barcode was 962 for COI and 1956 for 18S. Regions correspond to Outer Hauraki Gulf (Outer), Inner Hauraki Gulf (Inner) and Firth of Thames (Firth). Some samples had low number of reads and were therefore excluded from the diversity statistics calculation for 16S and COI.

			COI		18S	
Sample	Region	Season	reads	Sh-α	reads	Sh-α
C34	Outer	Cool	6315	52	2363	2
C35	Outer	Warm	4019	45	13765	2
C36	Outer	Cool	4720	83	7407	1
C37	Inner	Cool	2072	64	3051	3
CS8	Inner	Warm	2695	20	2223	6
C39	Inner	Cool	961	42	2199	2
C40	Firth	Cool	6467	17	5509	2
C41	Inner	Cool	7028	19	1956	3
C42	Outer	Cool	2556	30	2880	5
C43	Inner	Warm	7993	10	4580	2
C44	Inner	Cool	3474	15	3845	5
C45	Inner	Warm	6383	17	3263	2
C46	Inner	Warm	6064	25	3476	4
C47	Outer	Warm	3967	34	2287	3
C48	Outer	Cool	4723	39	3973	2
C49	Outer	Warm	414	-	5427	4
C50	Outer	Cool	7347	12	4044	2
C51	Outer	Warm	5579	53	5869	1
C52	Firth	Cool	10852	10	4513	3
C53	Inner	Cool	15235	11	5842	2
C54	Outer	Cool	6566	51	4209	3
C55	Firth	Warm	14111	25	4142	6
C56	Inner	Warm	3756	11	9373	4
C57	Outer	Warm	20124	14	6011	9

Table S4: The number of reads, Shannon (Sh-α) and Simpson's (Si-α) α diversity calculated per sample for whale scat. Diversity statistics were calculated by subsampling to the lowest number of reads per sample per DNA barcode: 1956 reads for 18S, 952 reads for COI. Regions correspond to Outer Hauraki Gulf (Outer) and Inner Hauraki Gulf (Inner). Not all samples had sampling location information (marked by N/A).

Scat sample	Region	Season	18S reads	Sh-α	COI reads	Sh-α
S01	Inner	Cool	-	-	-	-
S02	N/A	Cool	137094	4	675	-
S03	Inner	Cool	109592	6	5983	22
S04	Inner	Warm	39640	5	11796	12
S05	Outer	Warm	15258	3	18207	34
S06	Inner	Cool	11477	8	2075	37
S07	Inner	Cool	76565	3	14836	81
S08	Inner	Cool	92697	6	15136	29
S09	Inner	Cool	64078	4	4495	66
S10	Outer	Cool	-	-	8630	39
S11	Outer	Cool	-	-	4099	9
S12	Outer	Cool	-	-	26070	34
S13	Outer	Warm	-	-	73508	29
S14	Outer	Cool	7127	5	7763	52
S15	Outer	Cool	21132	5	17563	46
S16	Inner	Cool	227	-	6235	14
S17	N/A	Warm	6022	3	13606	25
S18	Inner	Warm	46235	2	-	-
S19	Inner	Warm	21677	11	-	-
S20	Inner	Cool	513	-	-	-

Table S5: P - value for each factor in multi-factorial PERMANOVA analyses used to investigate the difference in whale scat and zooplankton community composition due to sampling region and temperature season, and sample type (Type). Matrix indicates what distance matrix the PERMANOVA was based on and include: Jaccard similarity measure on presence-absence transformed data (Jaccard) or a Bray-Curtis similarity matrix on fourth root transformed data (Bray-Curtis), weighted (Unifrac - W) and unweighted UniFrac (Unifrac - U) distances. The CAP results for those comparisons that were significant for type can be found in Supplementary Material 2.

DNA barcode	Matrix	Scat samples	Plankton samples	Region	Season	Type
COI	Jaccard	14	23	0.002	<0.001	0.001
COI	Bray-Curtis	14	23	0.002	<0.001	<0.001
COI	Unifrac-U	14	23	0.002	<0.001	<0.001
COI	Unifrac-W	14	23	0.350	0.057	0.216
18S	Jaccard	11	24	0.009	0.042	<0.001
18S	Bray-Curtis	11	24	0.006	0.016	<0.001
18S	Unifrac-U	11	24	0.008	0.046	0.001
18S	Unifrac-W	11	24	0.007	<0.001	0.201

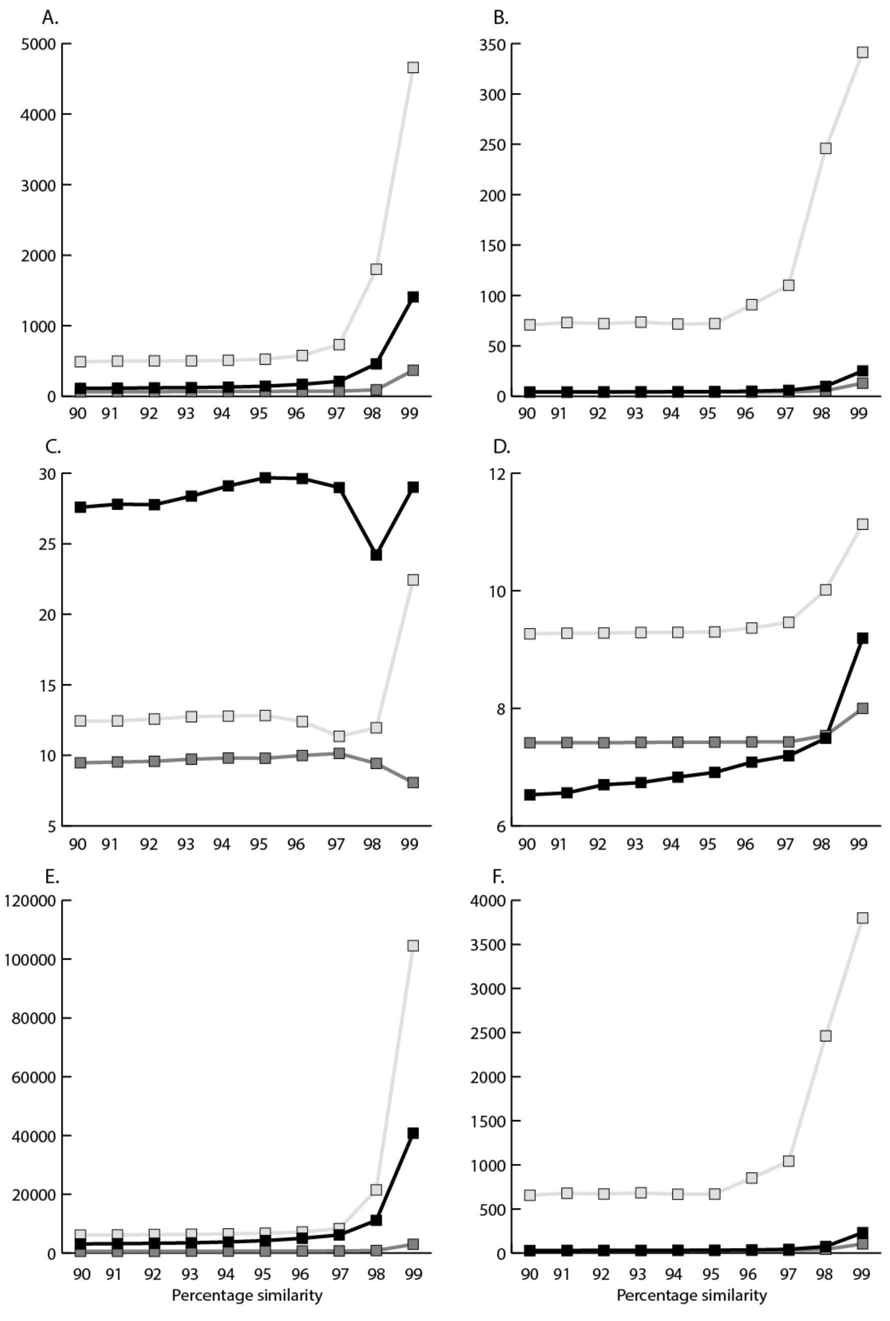

Figure S1: Change in the biodiversity statistics of each DNA barcode of Jost (2006, 2007) with change in clustering (90 – 99%) for the zooplankton community samples. Shown are effective α (Panel A), β (C), γ (E) and Shannon α (B), β (D), γ (F) diversity statistics.

Supplementary Material 1.2: Availability of data and reconstructing principal coordinate analyses (PCO) and canonical analyses of principal components (CAP)

Both unconstrained PCO and constrained CAP analyses were conducted on the zooplankton data for both DNA barcodes, using four different transformations reflecting OTU composition and relative read abundance, using OTU identity (Jaccard and Bray Curtis distances) and genetic similarity (UniFrac distances). PCO analyses are an unconstrained ordination that was conducted to visualise the data and show any emergent patterns. CAP analyses are constrained ordination that are conducted to test specific hypotheses [7].

Instead of putting all the visualisations into the Supplementary Material, we provide the data and R code for reconstructing both the transformations, PCO and CAP analyses on github:
[*github link to be added upon acceptance*]

This is also done for the whale scat for the PCO analyses:
[*github link to be added upon acceptance*]

Supp Mat References

1. O'Rorke R, Lavery S, Chow N, Takeyama H, Tsai P, Beckley LE, et al. Determining the diet of larvae of western rock lobster (*Panulirus cygnus*) using high-throughput DNA sequencing techniques. PLoS One. 2012;7: e42757. doi:10.1371/journal.pone.0042757
2. Leray M, Yang JY, Meyer CP, Mills SC, Agudelo N, Ranwez V, et al. A new versatile primer set targeting a short fragment of the mitochondrial COI region for metabarcoding metazoan diversity: application for characterizing coral reef fish gut contents. Front Zool. Frontiers in Zoology; 2013;10: 34–48. doi:10.1186/1742-9994-10-34
3. Jost L. Entropy and diversity. Oikos. 2006;113: 363–375.
4. Jost L. Partitioning diversity into independent alpha and beta components. Ecology. 2007;88: 2427–2439. doi:10.1073/pnas.0807060105
5. Charney N, Record S. vegetarian: Jost diversity measures for community data. In: Accessed from https://cran.r-project.org/web/packages/vegetarian/. 2012.
6. Drummond AJ, Newcomb RD, Buckley TR, Xie D, Dopheide A, Potter BC, et al. Evaluating a multigene environmental DNA approach for biodiversity assessment. GigaScience; 2015;4: 46. doi:10.1186/s13742-015-0086-1
7. Anderson MJ, Willis TJ. Canonical analysis of principal coordinates: A useful method of constrained ordination for ecology. Ecology. 2003;84: 511–525.

Supplementary Material 2: Faith's phylogenetic distance measure analysis and CAP analyses results.

Faith's phylogenetic distance measure for 18S DNA barcode for zooplankton community samples

Sample	ntaxa	pd.obs	pd.rand.mean	pd.rand.sd	pd.obs.rank	pd.obs.z	pd.obs.p
C34	15	2.46	2.58	0.80	582	-0.14	0.58
C35	20	3.92	3.22	0.93	842	0.76	0.84
C36	11	1.92	2.03	0.67	532	-0.16	0.53
C37	21	3.47	3.38	0.94	715	0.10	0.72
C38	37	5.30	5.06	1.13	703	0.21	0.70
C39	18	3.40	2.99	0.87	826	0.47	0.83
C40	15	2.72	2.60	0.80	741	0.15	0.74
C41	16	3.62	2.77	0.87	875	0.98	0.88
C42	38	4.79	5.18	1.13	497	-0.34	0.50
C43	20	3.85	3.22	0.92	846	0.69	0.85
C44	19	3.82	3.15	0.91	838	0.74	0.84
C45	22	4.05	3.48	0.97	821	0.60	0.82
C46	26	4.30	3.93	1.00	792	0.37	0.79
C47	16	2.71	2.73	0.80	637	-0.02	0.64
C48	18	2.77	2.97	0.86	527	-0.23	0.53
C49	42	4.40	5.50	1.13	119	-0.98	0.12
C50	26	3.69	3.94	1.01	547	-0.25	0.55
C51	15	2.73	2.61	0.80	741	0.15	0.74
C52	35	5.59	4.86	1.10	759	0.66	0.76
C53	21	3.65	3.35	0.92	793	0.32	0.79
C54	26	3.30	3.94	1.02	281	-0.62	0.28
C55	25	4.35	3.83	0.99	786	0.53	0.79
C56	22	4.10	3.45	0.93	838	0.70	0.84
C57	41	5.48	5.40	1.13	682	0.07	0.68

Faith’s phylogenetic distance measure for 18S DNA barcode for whale scat samples

Sample	ntaxa	pd.obs	pd.rand.mean	pd.rand.sd	pd.obs.rank	pd.obs.z	pd.obs.p
P14	37	5.35	5.03	1.10	733	0.29	0.73
P15	48	4.56	6.06	1.18	21	-1.27	0.02
P17	20	5.01	3.25	0.94	897	1.87	0.90
P18	8	2.75	1.60	0.61	947	1.89	0.95
P19	36	2.86	4.93	1.06	1	-1.96	0.00
P02	31	4.13	4.41	1.05	546	-0.27	0.55
P03	26	3.48	3.89	0.97	400	-0.43	0.40
P04	37	5.25	5.02	1.07	724	0.21	0.72
P05	38	4.33	5.12	1.09	244	-0.73	0.24
P06	45	5.47	5.83	1.17	533	-0.31	0.53
P07	32	2.89	4.52	1.05	4	-1.55	0.00
P08	46	5.26	5.88	1.17	411	-0.53	0.41
P09	20	2.62	3.20	0.87	225	-0.66	0.23

Faith’s phylogenetic distance measure for COI DNA barcode for zooplankton community samples

Sample	ntaxa	pd.obs	pd.rand.mean	pd.rand.sd	pd.obs.rank	pd.obs.z	pd.obs.p
P03	79	11.06	27.22	2.29	1	-7.06	0.001
P04	30	8.01	12.71	1.56	2	-3.02	0.002
P05	67	4.12	23.89	2.17	1	-9.10	0.001
P06	87	9.04	29.35	2.38	1	-8.55	0.001
P07	160	10.25	47.36	2.86	1	-12.99	0.001
P08	87	7.35	29.29	2.42	1	-9.08	0.001
P09	153	26.44	45.48	2.88	1	-6.61	0.001
P10	89	15.13	29.88	2.47	1	-5.97	0.001
P11	71	8.60	25.07	2.24	1	-7.34	0.001
P12	110	10.18	35.26	2.61	1	-9.62	0.001
P13	83	7.27	28.37	2.43	1	-8.67	0.001
P14	105	21.23	34.09	2.63	1	-4.89	0.001
P15	98	9.32	32.10	2.60	1	-8.77	0.001
P16	40	21.07	15.97	1.81	997	2.82	0.997

Faith's phylogenetic distance measure for COI DNA barcode for whale scat samples

Zooplankton	ntaxa	pd.obs	pd.rand.mean	pd.rand.sd	pd.obs.rank	pd.obs.z	pd.obs.p
C34	122	12.56	38.19	2.82	1	-9.09	0.001
C35	91	10.04	30.37	2.47	1	-8.22	0.001
C36	167	13.16	48.95	3.23	1	-11.08	0.001
C37	145	15.61	44.03	2.82	1	-10.10	0.001
C38	66	8.26	23.66	2.17	1	-7.10	0.001
C39	110	8.04	35.33	2.72	1	-10.03	0.001
C40	48	6.28	18.41	1.97	1	-6.15	0.001
C41	77	8.72	26.66	2.30	1	-7.79	0.001
C42	105	13.16	34.04	2.59	1	-8.05	0.001
C43	34	2.47	14.01	1.63	1	-7.08	0.001
C44	59	4.66	21.67	2.15	1	-7.93	0.001
C45	67	9.11	23.89	2.26	1	-6.55	0.001
C46	68	7.35	24.16	2.15	1	-7.82	0.001
C47	75	7.79	26.12	2.31	1	-7.92	0.001
C48	100	11.07	32.72	2.64	1	-8.19	0.001
C50	55	9.44	20.55	2.05	1	-5.43	0.001
C51	124	8.96	38.82	2.73	1	-10.93	0.001
C52	65	8.79	23.26	2.15	1	-6.74	0.001
C53	74	7.93	25.89	2.31	1	-7.77	0.001
C54	134	8.03	41.30	2.89	1	-11.51	0.001
C55	58	3.31	21.29	2.01	1	-8.95	0.001
C56	40	2.87	15.94	1.71	1	-7.64	0.001
C57	49	5.26	18.70	1.88	1	-7.14	0.001

CAP analysis results for 18S DNA barcode

Sample	Type	Region	Season	Temp	Jaccard - CAP1Type	Jaccard - CAP1Temp	Jaccard - CAP1R	Jaccard - CAP2R	Bray - CAP1Type	Bray - CAP1Temp
S14	Pooh	Outer	Spring	Cool	-0.76	0.78	0.26	0.17	-0.60	1.02
S15	Pooh	Outer	Spring	Cool	-1.84	0.56	-0.31	0.37	-1.88	0.66
S18	Pooh	Inner	Summer	Warm	-0.27	0.87	-0.39	0.25	0.10	1.35
S19	Pooh	Inner	Summer	Warm	-1.89	1.18	-0.26	1.36	-1.92	1.10
S03	Pooh	Inner	Winter	Cool	-0.49	-1.15	-0.33	0.99	-0.44	-0.98
S04	Pooh	Inner	Summer	Warm	-1.67	0.98	-0.98	1.27	-1.56	0.66
S05	Pooh	Outer	Summer	Warm	-1.71	0.97	-0.71	0.24	-1.69	0.60
S06	Pooh	Inner	Spring	Cool	-0.25	-0.64	0.59	-0.20	-0.13	-0.79
S07	Pooh	Inner	Spring	Cool	-1.77	-0.36	-1.23	0.77	-1.89	-0.61
S08	Pooh	Inner	Spring	Cool	-1.30	0.22	-0.17	1.37	-1.12	0.00
S09	Pooh	Inner	Winter	Cool	-0.07	-1.27	-0.65	0.64	-0.22	-1.24
C34	Plankton	Outer	Winter	Cool	0.65	-0.84	-1.22	-1.00	0.50	-0.52
C35	Plankton	Outer	Autumn	Warm	0.60	0.93	0.18	-0.84	0.67	0.80
C36	Plankton	Outer	Winter	Cool	0.29	-1.60	-1.24	-1.31	0.06	-1.62
C37	Plankton	Inner	Winter	Cool	0.26	-1.07	-0.89	0.85	-0.01	-1.08
C38	Plankton	Inner	Autumn	Warm	0.50	1.63	0.50	0.70	0.43	1.15
C39	Plankton	Inner	Winter	Cool	0.31	-1.00	-1.08	0.76	0.05	-1.01
C40	Plankton	Firth	Winter	Cool	0.76	-0.35	2.46	-1.51	0.85	-0.42
C41	Plankton	Inner	Winter	Cool	0.85	-0.51	1.16	0.38	0.95	-0.30
C42	Plankton	Outer	Winter	Cool	0.72	-0.62	0.81	-1.06	0.79	-0.59
C43	Plankton	Inner	Summer	Warm	0.74	1.24	1.10	0.57	0.70	1.68
C44	Plankton	Inner	Spring	Cool	0.80	-0.14	1.12	-0.12	0.90	0.24
C45	Plankton	Inner	Summer	Warm	0.67	1.27	0.86	0.93	0.60	1.50
C46	Plankton	Inner	Autumn	Warm	0.42	1.73	1.25	1.14	0.56	1.71

C47	Plankton	Outer	Summer	Warm	0.26	-0.59	-1.64	-1.11	0.17	-1.00
C48	Plankton	Outer	Spring	Cool	0.20	-1.25	-1.50	-1.29	0.04	-1.15
C49	Plankton	Outer	Summer	Warm	-1.31	1.10	-0.56	-0.55	-1.47	0.81
C50	Plankton	Outer	Spring	Cool	0.89	-0.53	0.42	-1.95	1.16	-0.54
C51	Plankton	Outer	Autumn	Warm	0.21	-0.64	-1.24	0.56	0.03	-0.83
C52	Plankton	Firth	Spring	Cool	0.72	-0.36	2.02	-1.44	0.91	-0.55
C53	Plankton	Inner	Spring	Cool	0.62	-1.52	-0.28	0.57	0.64	-1.42
C54	Plankton	Outer	Spring	Cool	0.54	-1.18	-1.01	-1.36	0.54	-1.21
C55	Plankton	Firth	Autumn	Warm	0.71	0.98	2.09	-0.10	0.74	1.16
C56	Plankton	Inner	Autumn	Warm	0.87	1.11	1.14	0.63	0.93	1.30
C57	Plankton	Outer	Autumn	Warm	0.75	0.05	-0.27	-0.69	0.61	0.10

Bray - CAP1R	Bray - CAP2R	UUF - CAP1Type	UUF - CAP1Temp	UUF - CAP1R	UUF - CAP2R
0.46	0.75	-0.22	0.84	0.56	-0.24
-0.33	0.70	-1.75	0.11	-0.04	0.84
-0.50	0.83	0.02	0.82	-0.82	0.49
-0.30	2.18	-1.61	0.44	-0.03	1.40
-0.36	0.47	-0.42	-0.65	0.35	1.01
-1.25	0.66	-1.26	1.17	-0.55	1.39
-0.57	0.43	-1.39	0.06	-0.14	0.39
0.75	-0.21	-0.15	-1.07	0.56	-1.28
-2.01	-0.12	-1.98	-1.12	-1.37	-0.04
-0.03	1.20	-1.03	0.52	0.34	1.15
-0.92	0.02	-0.06	-1.01	-0.31	0.24
-1.24	-1.19	0.67	-0.61	-1.10	-1.15

0.38	-1.20	0.57	0.49	-0.02	-0.02
-1.61	-1.29	0.09	-1.08	-1.57	-1.60
-1.03	0.45	0.09	-0.23	-0.07	1.46
0.81	0.89	0.59	1.23	0.67	0.64
-1.21	0.43	0.07	-0.13	-0.11	1.29
2.47	-1.50	0.86	-1.37	1.53	-2.87
1.14	0.03	0.54	-0.99	0.85	-0.28
1.24	-1.31	0.72	-0.26	1.03	-0.39
1.05	1.60	0.92	0.94	0.55	-0.15
1.34	0.22	0.28	0.14	0.76	0.53
0.71	1.54	0.59	1.19	0.47	0.90
1.42	1.54	0.12	1.25	0.97	0.56
-1.71	-0.61	0.03	-0.14	-2.00	-0.91
-1.57	-1.26	-0.16	-0.85	-1.62	-0.42
-0.57	-0.63	-0.61	-0.18	-0.76	-2.29
1.04	-2.50	1.09	-1.30	-0.34	-1.77
-1.47	0.05	-0.16	0.52	-0.72	1.56
2.02	-1.43	0.37	-0.09	1.63	-1.27
-0.46	-0.40	0.41	-1.57	0.33	0.30
-0.87	-1.59	0.69	0.20	-1.25	-0.56
1.92	0.51	0.41	0.54	1.59	0.59
1.35	0.90	0.85	1.36	0.77	0.45
-0.09	-0.14	0.81	0.81	-0.14	0.02

CAP analysis results for COI DNA barcode

Sample	Type	Region	Season	Temp	Jaccard - CAP1Type	Jaccard - CAP1Temp	Jaccard - CAP1R	Jaccard - CAP2R	Bray - CAP1Type	Bray - CAP1Temp

P03	Pooh	Inner	Winter	Cool	-0.96	-0.97	-0.08	-1.16	-0.89	-0.90
P04	Pooh	Inner	Summer	Warm	-0.43	0.36	-0.57	0.04	-0.31	0.00
P05	Pooh	Outer	Summer	Warm	-2.18	-0.72	1.16	0.19	-2.22	-0.75
P06	Pooh	Inner	Spring	Cool	-0.24	-0.86	-0.14	-0.97	-0.21	-0.87
P07	Pooh	Inner	Spring	Cool	-1.36	-1.09	1.35	-1.08	-1.46	-1.02
P08	Pooh	Inner	Spring	Cool	-1.00	-0.87	-0.17	-0.68	-0.87	-0.96
P09	Pooh	Inner	Winter	Cool	-0.39	-0.68	0.44	-1.40	-0.31	-0.66
P10	Pooh	Outer	Spring	Cool	-2.02	-0.68	1.05	0.26	-2.15	-0.67
P11	Pooh	Outer	Spring	Cool	-0.99	-0.81	1.07	0.33	-0.92	-0.79
P12	Pooh	Outer	Spring	Cool	-1.39	-0.92	1.75	0.50	-1.35	-0.83
P13	Pooh	Outer	Summer	Warm	-0.32	0.03	1.11	-0.28	-0.30	-0.13
P14	Pooh	Outer	Spring	Cool	0.04	0.26	-0.39	0.27	0.21	0.36
P15	Pooh	Outer	Spring	Cool	-1.93	-0.98	1.29	0.31	-1.96	-0.94
P16	Pooh	Inner	Spring	Cool	-0.75	-0.15	-0.34	-1.09	-0.77	-0.03
C34	Plankton	Outer	Winter	Cool	0.12	-0.45	1.06	0.14	0.01	-0.44
C35	Plankton	Outer	Autumn	Warm	0.49	0.11	0.54	1.37	0.46	-0.14
C36	Plankton	Outer	Winter	Cool	0.12	-0.49	1.24	-0.17	0.01	-0.52
C37	Plankton	Inner	Winter	Cool	-0.07	-0.89	0.21	-1.11	-0.17	-0.78
C38	Plankton	Inner	Autumn	Warm	1.17	1.33	-1.61	-0.70	1.23	1.33
C39	Plankton	Inner	Winter	Cool	0.00	-0.75	0.16	-0.94	-0.13	-0.73
C40	Plankton	Firth	Winter	Cool	0.58	-0.28	-1.46	2.72	0.51	-0.37
C41	Plankton	Inner	Winter	Cool	0.71	-0.16	-1.16	-0.51	0.69	-0.17
C42	Plankton	Outer	Winter	Cool	0.42	-0.13	-0.40	0.31	0.45	-0.07
C43	Plankton	Inner	Summer	Warm	1.04	2.42	-1.12	-1.03	1.14	2.61
C44	Plankton	Inner	Spring	Cool	0.49	-0.23	-0.33	0.32	0.44	-0.34
C45	Plankton	Inner	Summer	Warm	1.11	2.13	-1.27	-0.59	1.23	2.28

C46	Plankton	Inner	Autumn	Warm	1.41	2.02	-1.54	0.14	1.49	2.04
C47	Plankton	Outer	Summer	Warm	0.18	-0.08	1.20	1.08	0.09	-0.22
C48	Plankton	Outer	Spring	Cool	-0.12	-0.62	1.25	0.99	-0.20	-0.56
C50	Plankton	Outer	Spring	Cool	0.42	-0.51	0.22	1.16	0.37	-0.55
C51	Plankton	Outer	Autumn	Warm	0.34	0.06	1.01	-0.01	0.25	-0.01
C52	Plankton	Firth	Spring	Cool	0.90	0.04	-1.76	2.38	0.89	-0.02
C53	Plankton	Inner	Spring	Cool	0.63	-0.52	-0.50	-0.30	0.64	-0.48
C54	Plankton	Outer	Spring	Cool	0.01	-0.98	1.01	-0.03	-0.01	-0.96
C55	Plankton	Firth	Autumn	Warm	1.44	2.22	-1.80	0.93	1.43	2.19
C56	Plankton	Inner	Autumn	Warm	1.40	2.21	-1.39	-0.77	1.47	2.28
C57	Plankton	Outer	Autumn	Warm	1.14	1.65	-1.07	-0.61	1.23	1.83

Bray - CAP1R	Bray - CAP2R	UUF - CAP1Type	UUF - CAP1Temp	UUF - CAP1R	UUF - CAP2R
-0.15	-1.15	-0.49	-1.00	-0.33	-1.07
-0.59	1.47	-1.01	0.33	-0.15	-1.07
1.26	-0.19	-1.38	0.23	1.10	0.31
-0.10	-1.22	-0.58	-0.86	-0.32	-1.24
1.70	-1.33	-1.02	-0.94	0.85	-1.57
-0.31	-0.08	-0.49	-0.54	-0.15	-1.13
0.63	-1.93	-1.08	-1.01	0.29	-1.25
1.17	-0.13	-1.43	-0.56	0.90	0.21
1.06	0.95	-0.84	-0.50	1.15	0.32
1.98	0.43	-1.12	-0.44	1.61	0.47
1.21	-1.15	-0.49	0.45	1.28	0.53
-0.68	0.33	-0.97	-0.45	0.20	-0.18

1.39	0.22	-1.27	-0.88	1.03	-0.49
-0.32	-1.46	-1.28	-0.61	-0.05	-1.08
1.09	-0.31	-0.04	-0.33	1.30	0.76
0.43	2.50	0.42	0.60	0.39	1.14
1.31	-1.05	0.16	-0.53	1.21	0.07
0.30	-0.87	0.17	-0.91	-0.15	-1.10
-1.80	-0.94	0.85	0.89	-1.10	-0.40
0.24	-0.60	0.37	-0.27	-0.07	-0.84
-1.36	3.05	0.71	0.49	-1.37	1.52
-1.19	-0.65	0.78	-0.67	-0.93	-0.01
-0.61	0.49	0.42	-0.80	-0.45	0.09
-1.21	-1.51	0.54	1.71	-1.06	-0.65
-0.26	0.74	0.52	0.46	-0.63	-0.43
-1.34	-0.93	0.58	1.31	-0.45	-0.46
-1.59	0.16	1.14	1.59	-0.85	0.34
1.32	1.67	0.27	0.76	0.95	0.84
1.27	2.00	0.29	-0.38	0.90	0.79
-0.04	2.02	0.59	-0.82	0.24	1.38
0.92	-0.67	0.62	1.08	0.53	0.44
-1.74	2.45	0.92	-0.34	-1.69	2.07
-0.48	-0.03	0.82	-0.39	-0.88	0.46
0.99	0.12	0.43	-0.98	0.57	0.22
-1.71	0.39	1.19	1.51	-2.18	1.88
-1.46	-1.06	0.87	1.83	-1.01	-0.44
-1.33	-1.73	0.84	0.96	-0.69	-0.44

Supplementary Material 3: Matched water sample collection supplementary material

Introduction

We collected matched water control samples alongside the scat samples to check that the dominant taxa in the scat samples are attributable to the whale scat and not environmental or laboratory contamination.

Collection

Matched water samples were collected, using the same method, from water adjacent to the whale scat immediately after sample collection.

Characterising composition of matched water control samples

This was done in the same way as described for the plankton and whale scat samples in the main manuscript.

Investigating the impact of environmental contamination on whale scat samples

To determine environmental contamination on the scat samples we used the dataset of matched water samples collected concurrently to a subset of the scat samples. For these paired samples, we first identified the OTUs at high abundance (arbitrary >1% or >5%) in the matched water as potential environmental contaminants in the scat sample. These OTUs were removed from the scat samples to check potential environmental contamination, giving a new sample type – 'control' whale scat sample. We constructed PCO graphs to visualise the clustering of scat and matched water samples and then control scat and matched water samples.

If the control scat sample differs significantly from its paired matched water where the standard scat samples are not, this suggests that environmental contamination is a factor in the composition of the scat samples. Alternatively, similarity could exist because matched water samples were taken predominantly in areas where whales were foraging. Therefore, the matched water sample could reflect the taxa in a potential prey patch. To test this hypothesis, we constructed similarity matrices as described above, for both composition and relative abundance of taxa, for both the COI and 18S DNA barcodes. We then repeated the PERMANOVA with sample type, region and season as fixed factors for all matched water and scat samples (raw and rarefied data), then paired matched water and control whale scat samples (raw and rarefied data). Due to the multiple comparisons, we applied the Bonferroni correction to the results within each DNA barcode ($\alpha = 0.05$; $\alpha/8 = 0.006$).

RESULTS

Characterising composition of matched water samples

All matched water samples produced reads for either COI and/or 18S DNA barcodes (Stable 3-1), although two matched water samples (M22 and M23) in the 18S DNA barcode were excluded from rarefaction-based analyses due to low number of reads. Chordata and Arthropoda were the most common Phyla observed across matched water samples and DNA barcodes (SFig. 3-1). We restricted our analyses to the 1,101 COI DNA barcode OTUs and the 267 18S DNA barcode OTUs identified to Class.

No evidence for substantial environmental contamination of whale scat samples

Principal coordinate analyses showed that the scat samples did not cluster distinctly from the matched water controls for the COI and 18S DNA barcodes (Figs S3-2 and S3-3). Multi-factorial PERMANOVAs, incorporating season and region, showed sample type was not a significant factor for any dataset analysed (Table S3-2).

To examine whether this lack of differentiation was due to potential environmental contaminants in the scat, we removed OTUs abundant in the matched water samples from the paired scat samples to construct 'control' whale scat samples. Results were similar when excluded OTUs represented either 1% or 5% of matched water samples, therefore, we present results at the conservative 1% threshold. The control scat and matched water samples scattered throughout the PCO visualisation, with no obvious clustering by sample type (SFig 3-5 and 3-6). PERMANOVA analyses suggested that sample type was not a significant driver of differences in sample compositions, based on relative abundance, composition or phylogenetic similarity, for either the COI or 18S DNA barcodes. Results were non-significant using rarefied and full datasets, and with a limited dataset comparing the paired scat and adjacent water samples (STable 3-2).

Supplementary Table 3-1: The number of reads, Shannon (Sh-α) and Simpson's (Si-α) α diversity calculated per sample for whale scat (provided here for comparative purposes) and matched water (MW). Diversity statistics were calculated by subsampling to the lowest number of reads per sample per DNA barcode: 1956 reads for 18S, 952 reads for COI. Regions correspond to Outer Hauraki Gulf (Outer) and Inner Hauraki Gulf (Inner). Not all samples had sampling location information (marked by N/A).

Scat sample	Region	Season	18S reads	Sh-α	Si-α	COI reads	Sh-α	Si-α	MW sample	18S reads	Sh-α	Si-α	COI reads	Sh-α	Si-α
S01	Inner	Cool	-	-	-	-	-	-	M21	2	1	51	13092	62	36
S02	N/A	Cool	137094	4	2	675	-	-	M22	11	-	-	952	14	9
S03	Inner	Cool	109592	6	3	5983	22	9	M23	721	-	-			
S04	Inner	Warm	39640	5	3	11796	12	9	M24	2038	6	4	8093	2	2
S05	Outer	Warm	15258	3	2	18207	34	24	M25	71339	8	4			
S06	Inner	Cool	11477	8	4	2075	37	22	M26	7231	7	4	12210	57	35
S07	Inner	Cool	76565	3	2	14836	81	51	M27	17355	2	1	97630	14	6
S08	Inner	Cool	92697	6	4	15136	29	12	M28	134174	10	6			
S09	Inner	Cool	64078	4	2	4495	66	38	M29	16561	3	2	17069	30	14
S10	Outer	Cool	-	-	-	8630	39	25	M30	-	-	-	30953	43	24
S11	Outer	Cool	-	-	-	4099	9	4	M31	-	-	-	42074	47	28
S12	Outer	Cool	-	-	-	26070	34	19	M32	-	-	-	43752	60	37
S13	Outer	Warm	-	-	-	73508	29	14	M33	-	-	-	2168	45	25
S14	Outer	Cool	7127	5	3	7763	52	33							
S15	Outer	Cool	21132	5	2	17563	46	30							
S16	Inner	Cool	227	-	-	6235	14	10							
S17	N/A	Warm	6022	3	2	13606	25	14							
S18	Inner	Warm	46235	2	2										
S19	Inner	Warm	21677	11	8										
S20	Inner	Cool	513	-	-										

Supplementary Table 3-2: P-value for each factor in multi-factorial PERMANOVA used to investigate the impact of environmental contamination on the whale scat samples. For each analysis, the table shows the following: DNA barcode, dataset used, similarity matrix (Matrix), sample size for whale scat (scat samples), matched water control samples (MWC), control whale scat samples, and the p-value from the single-factor PERMANOVA where sample type was the factor. The dataset used was either rarefied (number of reads) or the raw data (Raw), using either all available samples (all) or only paired whale scat and matched water control samples (paired). Some samples were excluded from the rarefied dataset due to lower number of reads. These datasets were then transformed using a Jaccard similarity measure on presence-absence transformed data (Jaccard) or a Bray-Curtis similarity matrix on fourth root transformed data (Bray-Curtis), weighted (Unifrac-W) and unweighted unifrac (Unifrac-U) distances. The Bonferroni-corrected p-value is 0.006; no analyses met this criteria for factor 'Type".

DNA barcode	Dataset	Matrix	Scat samples	MWC samples	Control scat	Region	Season	Type
COI	Rarefied (952 reads) - all	Jaccard	14	9	-	0.045	0.290	0.126
COI	Rarefied (952 reads) - all	Bray-Curtis	14	9	-	0.057	0.307	0.134
COI	Rarefied (952 reads) - all	Unifrac-U	14	9	-	0.011	0.303	0.220
COI	Rarefied (952 reads) - all	Unifrac-W	14	9	-	0.186	0.160	0.266
COI	Raw – all	Jaccard	14	9	-	0.071	0.227	0.061
COI	Raw – all	Bray-Curtis	14	9	-	0.071	0.280	0.102
COI	Raw – all	Unifrac-U	14	9	-	0.001	0.200	0.149
COI	Raw – all	Unifrac-W	14	9	-	0.200	0.289	0.216
COI	Rarefied (952 reads) – paired	Jaccard	-	8	8	0.279	0.213	0.555
COI	Rarefied (952 reads) – paired	Bray-Curtis	-	8	8	0.110	0.063	0.203
COI	Rarefied (952 reads) – paired	Unifrac-U	-	8	8	0.015	0.068	0.593
COI	Rarefied (952 reads) - paired	Unifrac-W	-	8	8	0.280	0.213	0.555
COI	Raw – paired	Jaccard	-	8	8	0.084	0.049	0.115
COI	Raw – paired	Bray-Curtis	-	8	8	0.081	0.049	0.134
COI	Raw – paired	Unifrac-U	-	8	8	0.239	0.224	0.550
COI	Raw – paired	Unifrac-W	-	8	8	0.014	0.162	0.014
18S	Rarefied (1956) - all	Jaccard	11	7	-	0.212	0.264	0.724

18S	Rarefied (1956) - all	Bray-Curtis	11	7	-	0.217	0.389	0.502
18S	Rarefied (1956) - all	Unifrac-U	11	7	-	0.596	0.169	0.946
18S	Rarefied (1956) - all	Unifrac-W	11	7	-	0.903	0.452	0.560
18S	Raw – all	Jaccard	13	8	-	0.495	0.521	0.179
18S	Raw – all	Bray-Curtis	13	8	-	0.374	0.563	0.285
18S	Raw – all	Unifrac-U	13	8	-	0.684	0.365	0.363
18S	Raw – all	Unifrac-W	13	8	-	0.884	0.303	0.355
18S	Rarefied (1956) -paired	Jaccard	-	7	7	0.723	0.853	0.049
18S	Rarefied (1956) -paired	Bray-Curtis	-	7	7	0.831	0.900	0.032
18S	Rarefied (1956) -paired	Unifrac-U	-	7	7	0.771	0.427	0.175
18S	Rarefied (1956) -paired	Unifrac-W	-	7	7	0.739	0.888	0.046
18S	Raw – paired	Jaccard	-	7	7	0.497	0.868	0.039
18S	Raw – paired	Bray-Curtis	-	7	7	0.651	0.850	0.025
18S	Raw – paired	Unifrac-U	-	7	7	0.862	0.646	0.104
18S	Raw – paired	Unifrac-W	-	7	7	0.730	0.863	0.019

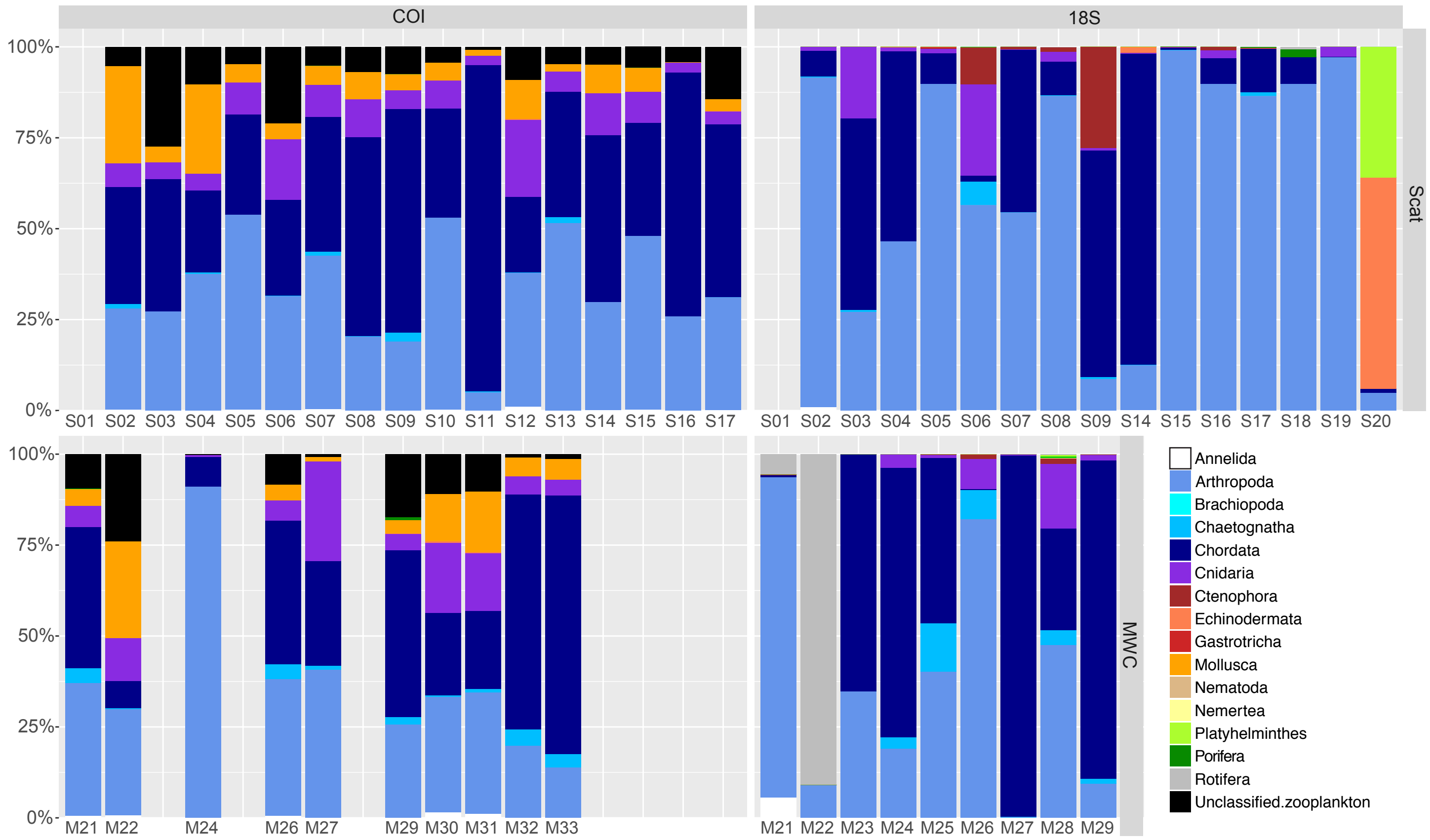

SFigure 3-1: Summary of reads by taxa for whale scat and matched water samples stratified by sample type and DNA barcodes. Sample name at bottom of bars reflects sample type (MW – M, and whale scat – S). The matched water sample for a given whale scat sample is directly below the whale scat sample e.g. 29 is the matched water for P09. Whale scat samples S14-S17 did not have matched waters and not all samples were successfully sequenced

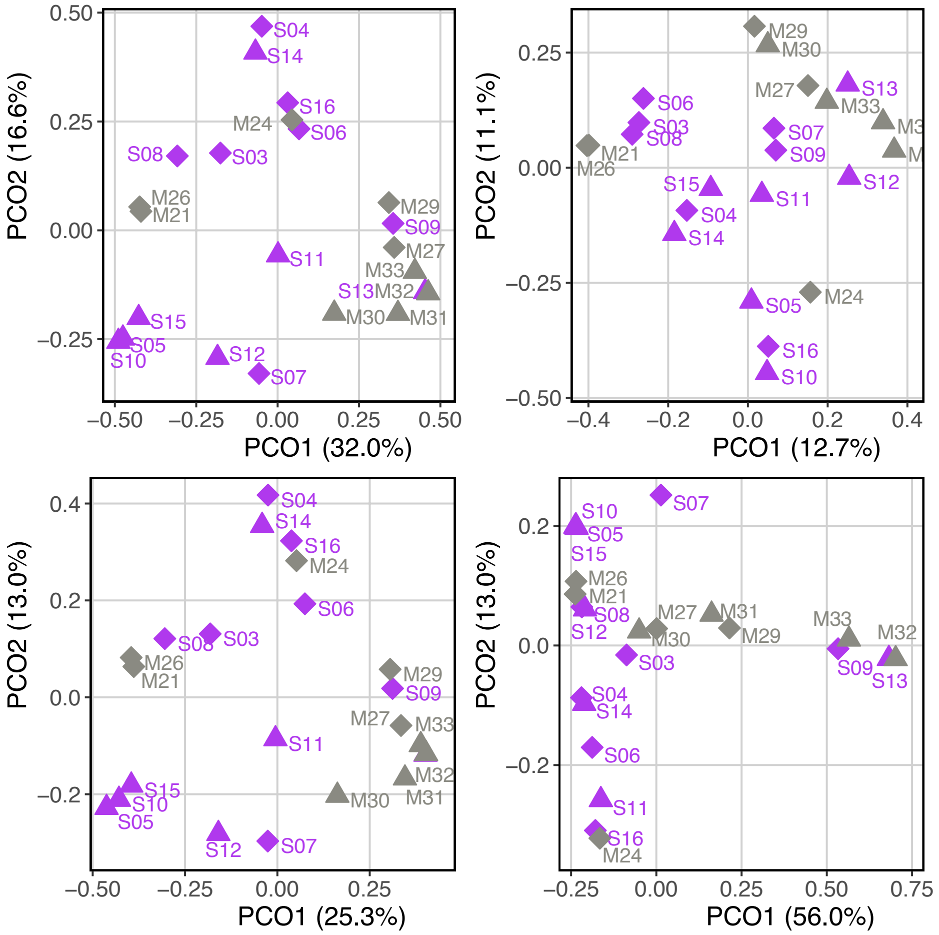

SFigure 3-2 Clustering of whale scat (purple) and matched water samples (grey) based on principal coordinate analysis (PCO) of the COI DNA barcode. PCO is based on different distance matrices, from the top left: Bray-Curtis distance matrix based on fourth root transformed data unweighted and weighted UniFrac distance matrices and Jaccard distance matrix based on presence/absence transformed data. Region is reflected by the shape, with diamond represent Inner Gulf samples and triangles representing Outer Gulf samples. Data were rarefied to 952 reads per sample.

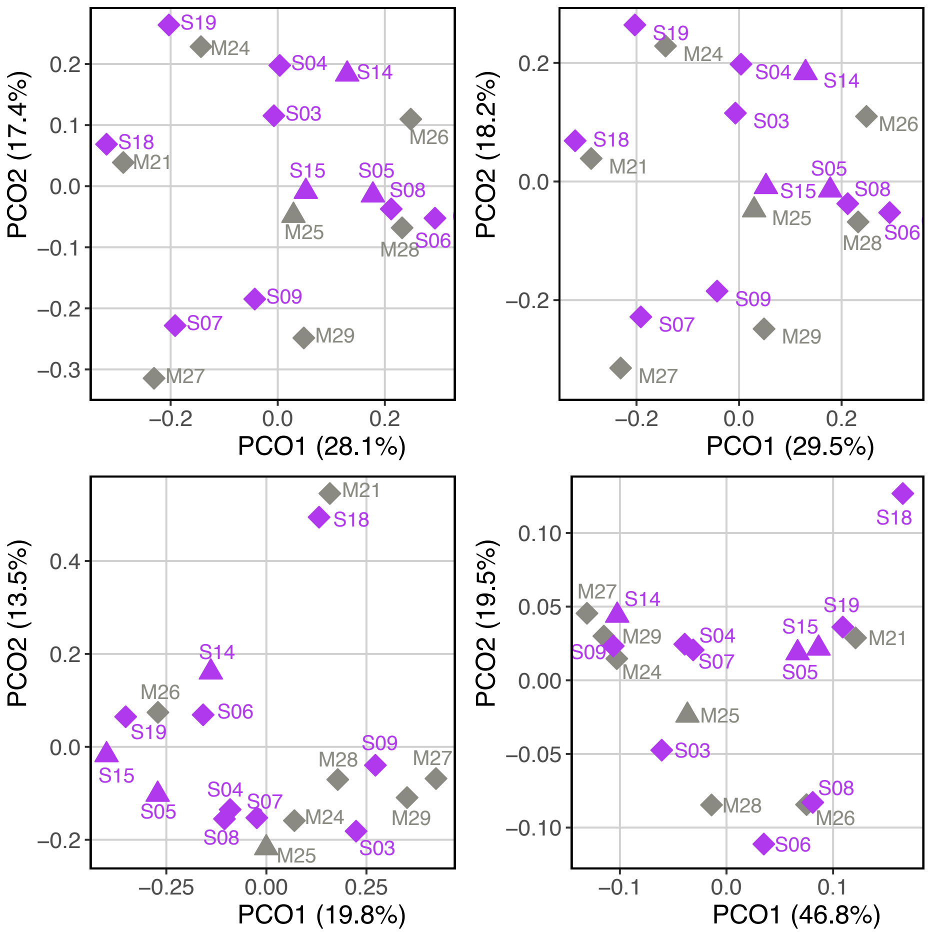

Supplementary Figure 3-3: Clustering of whale scat (purple) and matched water samples (grey) based on principal coordinate analysis (PCO) of the 18S DNA barcode. PCO is based on different distance matrices, from the top left: Bray-Curtis distance matrix based on fourth root transformed data, unweighted and weighted UniFrac distance matrices and Jaccard distance matrix based on presence/absence transformed data. Region is reflected by the shape, with diamond represent Inner Gulf samples and triangles representing Outer Gulf samples. Data were rarefied to 1956 reads per sample.

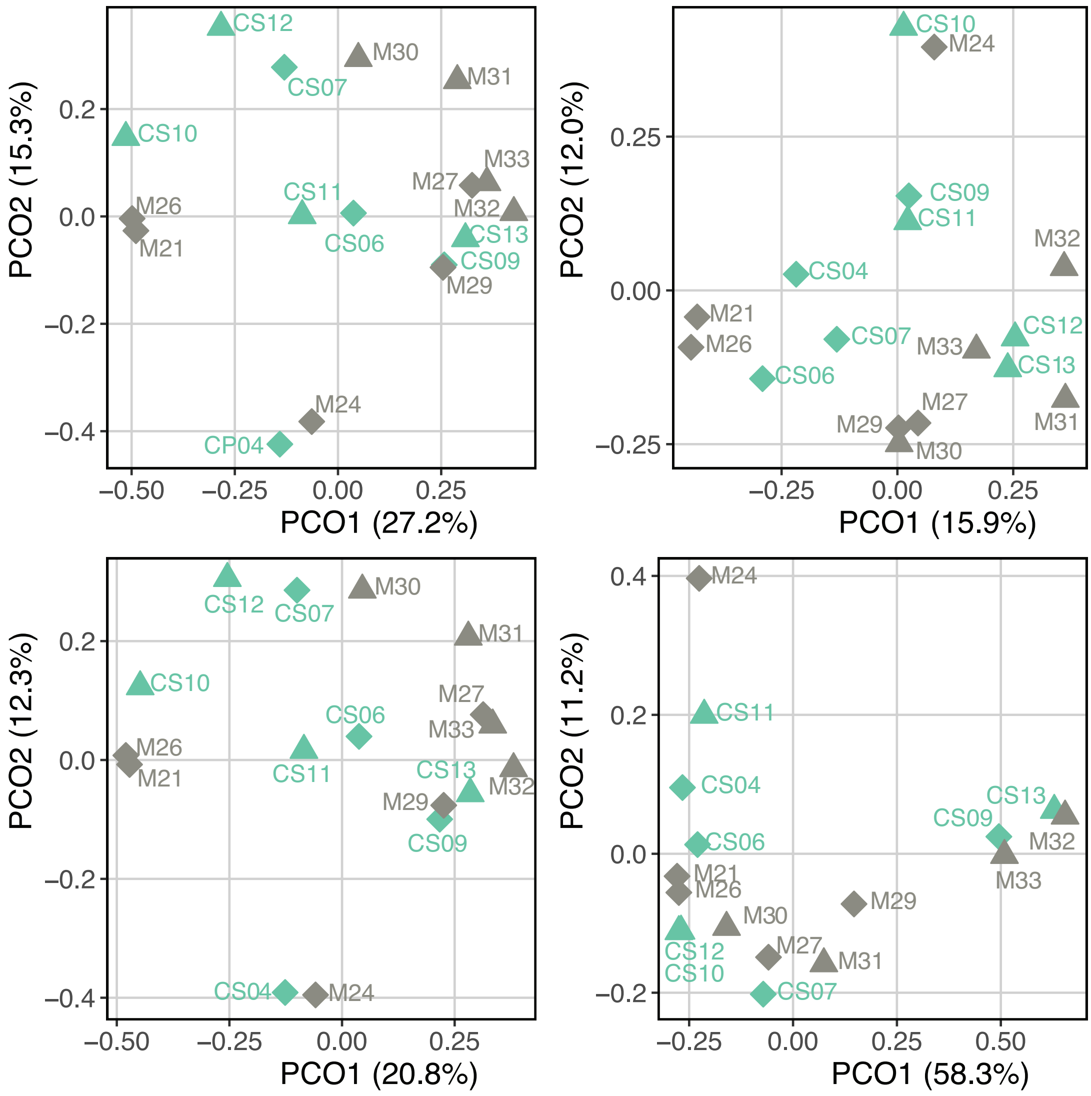

SFig 3-4: Clustering of control whale scat (aquamarine) and matched water samples (grey) based on principal coordinate analysis (PCO) of the COI DNA barcode. PCO is based on different distance matrices, from the top left: Bray-Curtis distance matrix based on fourth root transformed data, unweighted and weighted UniFrac distance matrices and Jaccard distance matrix based on presence/absence transformed data. Region is reflected by the shape, with diamond represent Inner Gulf samples and triangles representing Outer Gulf samples. Data were rarefied to 952 reads per sample.

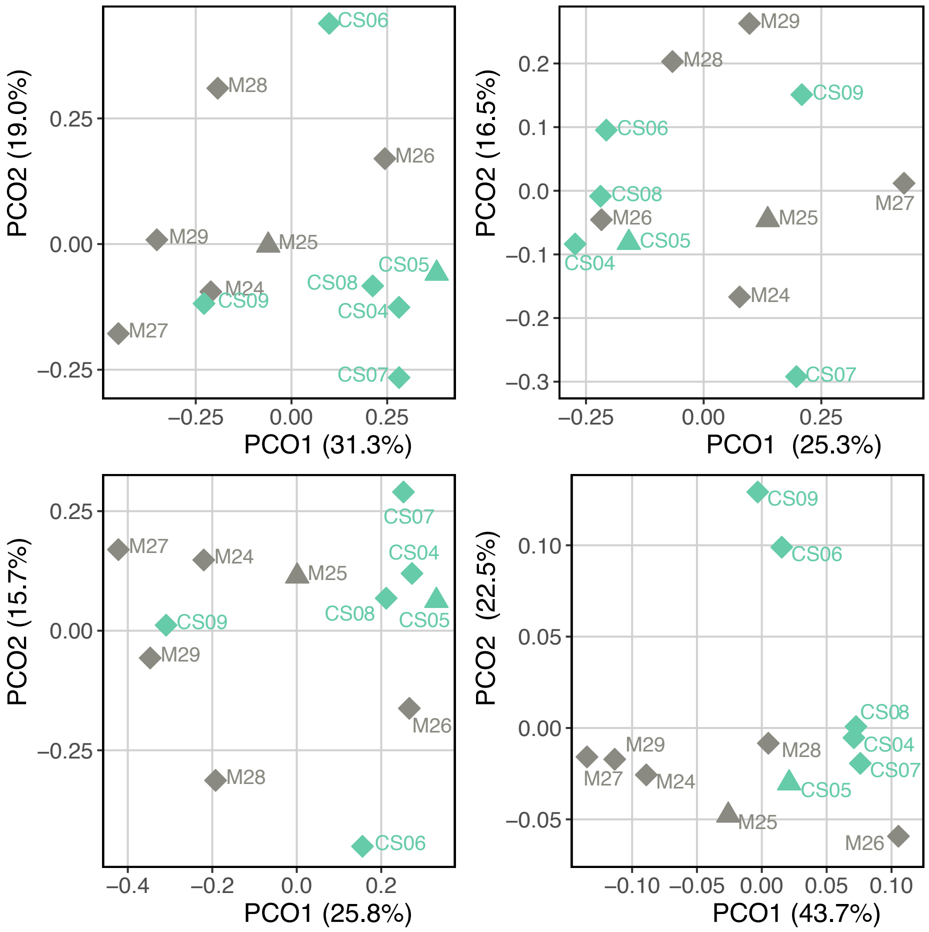

SFig 3-5: Clustering of control whale scat (aquamarine) and matched water samples (grey) based on principal coordinate analysis (PCO) of the 18S DNA barcode. PCO is based on different distance matrices, from the top left: Bray-Curtis distance matrix based on fourth root transformed data, unweighted and weighted UniFrac distance matrices and Jaccard distance matrix based on presence/absence transformed data. Region is reflected by the shape, with diamond represent Inner Gulf samples and triangles representing Outer Gulf samples. Data were rarefied to 1956 reads per sample.
